# Genome‐Wide Analysis Successfully Resolves Population Structure Shaped by Recent Divergence in the Endangered Bagrid Catfish *Pseudobagrus ichikawai*

**DOI:** 10.1002/ece3.73263

**Published:** 2026-03-26

**Authors:** Keisuke Onuki, Ryoichi Tabata, Tappei Mishina, Mutsumi Nishida, Katsutoshi Watanabe

**Affiliations:** ^1^ Laboratory of Animal Ecology, Graduate School of Science Kyoto University Kyoto Japan; ^2^ Ecological Genetics Laboratory National Institute of Genetics Shizuoka Japan; ^3^ Lake Biwa Museum Shiga Japan; ^4^ Faculty of Agriculture, Kyushu University Fukuoka Japan; ^5^ Atmosphere and Ocean Research Institute The University of Tokyo Chiba Japan

**Keywords:** freshwater fish, historical demography, MIG‐seq, Nekogigi, phylogeography, whole genome resequencing

## Abstract

The population structure and history of the endangered bagrid catfish *Pseudobagrus ichikawai* were assessed to provide insights into its conservation through comparative analyses of several traditional genetic markers and genome‐wide SNP data. The species is distributed in the Ise Bay area, located in central Honshu Island, Japan, a biogeographically unique region with endemic freshwater species. Samples were collected across all major populations, and genetic differentiation, population history, and demographic trends were inferred. Genetic analyses were conducted using whole mitochondrial genome sequences (mitogenomes), microsatellite polymorphisms, reduced‐representation genome sequencing (MIG‐seq), and whole‐genome resequencing. For the latter two methods, the newly determined chromosome‐level genome assembly was used as a reference. Genome‐wide data revealed a pattern of population differentiation that could not be detected with mitogenome or microsatellite data. This differentiation pattern, including genetic similarity between populations across the eastern and western sides of Ise Bay (trans‐Ise Bay pattern), appears to reflect population connectivity facilitated by paleo‐river systems during the Last Glacial Period. Genetic diversity indices showed low variability across populations, suggesting historical bottlenecks and, for some populations, recent inbreeding. By utilizing genome‐wide data, this study elucidated the subtle population structure of *P. ichikawai* in unprecedented detail. This approach provides a deeper understanding of the species' population history under geographic and climatic influences, contributing to conservation strategies for regional biodiversity management. Our results also demonstrate that the absence of detectable population structure using traditional markers does not necessarily preclude its existence, highlighting the critical role of genome‐wide data in uncovering cryptic diversity.

## Graphical abstract

Using genome‐wide SNP data, we revealed previously undetected population structure in the endangered bagrid catfish *Pseudobagrus ichikawai* . Our results suggest that past paleo‐river systems shaped the current genetic connectivity across the Ise Bay area, while low genetic diversity indicates historical bottlenecks and recent inbreeding. These findings provide key insights into the species' evolutionary history and inform conservation strategies.

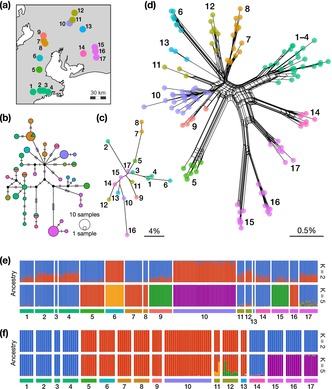

## 1 Introduction

Population structure is an important aspect for understanding the spatio‐temporal dynamics of biodiversity, including the formation of species distribution ranges, population dynamics, and their relationships to geography and climate changes (Avise et al. 1987; Avise 2000; Hewitt 2004a). For species of conservation concern, such information is needed not only for reconstructing their natural history but also for assessing genetic diversity and defining conservation units (Allendorf et al. 2022; Avise 1994; Fraser and Bernatchez 2001). Over the last four decades, genetic markers have played an essential role in achieving these objectives. In particular, animal mitochondrial DNA (mtDNA) has been widely used to elucidate population structure within species, resulting in a vast body of research (Avise 1994, 2000; Hewitt 2004b). To compensate for the limitations of mtDNA, primarily due to its maternal inheritance, various nuclear DNA markers such as microsatellites and fingerprinting markers (e.g., AFLP) have also been developed and broadly applied (Brito and Edwards 2009; Hewitt 2004b; Zhang and Hewitt 2003).

Over the past decade, whole‐genome sequencing and various reduced‐representation genome sequencing approaches have become widely used to address limitations such as the small number of loci, insufficient phylogenetic information, and high costs of development and experimentation (Brumfield et al. 2003; Morin et al. 2004; Zink 2010). These methods have enabled the acquisition of large‐scale genome‐wide polymorphism data using high‐throughput DNA sequencing technologies. Such data provide unprecedented insights into population and species history, including population differentiation, secondary contact, interspecific hybridization, and population size fluctuations, with high precision across a wide range of time scales (Edwards et al. 2015, 2022; McCormack et al. 2013; Nadachowska‐Brzyska et al. 2022; Nater et al. 2015). Based on these advanced approaches, reconstructing the spatio‐temporal structure of biodiversity, including conservation‐target species, has become a key challenge for modern phylogeography and conservation genetics (Edwards et al. 2022; Marske et al. 2013).

**FIGURE 1:**
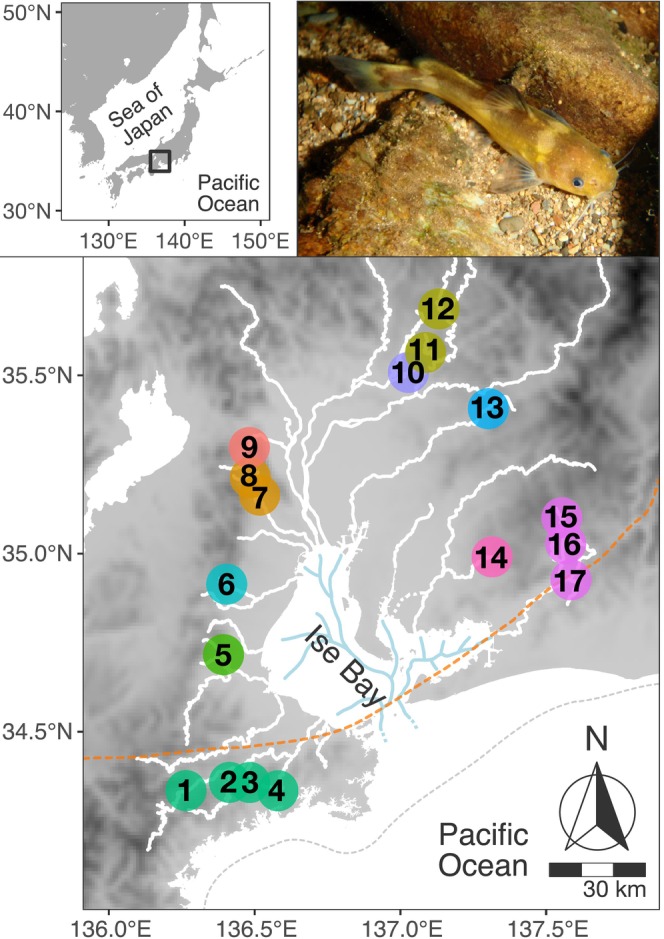
The Ise Bay area, where *Pseudobagrus ichikawai* is distributed, and the sampling sites in this study. The correspondence between the numbers on the map and the names of the sampling sites is as follows: (1) Miya‐01; (2) Miya‐02; (3) Miya‐03; (4) Miya‐04; (5) Kumozu; (6) Suzuka; (7) Inabe‐01; (8) Inabe‐02; (9) Ibi; (10) Nagara; (11) Kiso‐01; (12) Kiso‐02; (13) Shonai; (14) Yahagi; (15) Toyo‐01; (16) Toyo‐02; and (17) Toyo‐03. Sampling sites are color‐coded according to their respective river systems (see also Table 1). The white lines indicate the present‐day rivers (the dashed line represents the flow path before artificial river modification). The blue lines and the gray dashed line indicate the estimated paleo‐river systems and shoreline during the Last Glacial Maximum, respectively. The orange dashed line indicates the Median Tectonic Line, a major tectonic line extending across southwest Japan. Photo: *P. ichikawai* at the sampling site 10 in a tributary of the Nagara River system.

The bagrid catfish *Pseudobagrus ichikawai* (known as “Nekogigi” in Japanese; referred to as *Tachysurus ichikawai* in some recent literature) is an endangered freshwater fish species endemic to Japan. It is distributed only in the upper and middle reaches of rivers around Ise and Mikawa Bays in central Honshu Island (Mori and Nagoshi 2001; Okada and Kubota 1957; Figure 1). This region (hereafter referred to as the Ise Bay area), encompassing a catchment area of 18,135 km^2^, is a biogeographically distinctive area harboring unique aquatic and terrestrial biota. In addition to *P. ichikawai* , the Ise Bay area includes several other endemic freshwater fishes such as the minnow *Pseudorasbora pugnax*, the striped loach *Cobitis minamorii tokaiensis*, the nemacheilid loach *Lefua tokaiensis*, and the swamp goby *Rhinogobius telma* (Ito et al. 2019; Kawase and Hosoya 2015; Nakajima 2012; Suzuki et al. 2019; Watanabe et al. 2017). The topography of the shallow inner bay (average depth of 17 m) suggests that inflowing river systems were likely merged into one or a few paleo‐river systems during periods of marine regression (Figure 1). Suggestive of the influence of such paleo‐river systems, it has been observed that populations of the minnow *Pseudorasbora pugnax* and *Hemigrammocypris neglectus* (originally referred to as *H. rasborella* ), both of which typically inhabit floodplains, show a close genetic relationship between the eastern and western populations across the bay (trans‐Ise Bay distribution), although this pattern would not be expected from the current geography (Cho and Mukai 2023; Watanabe and Mori 2008). However, the population structure of *P. ichikawai* remains unclear, as it was found to be monomorphic across its distribution in the mtDNA control region, which is generally considered a hypervariable region in animal mtDNA (Watanabe and Nishida 2003). The low genetic diversity of this species has been an obstacle to clarify its fine‐scale population differentiation and conservation units and to assess the loss of genetic diversity. More sensitive genetic analyses are necessary to obtain fundamental information on the natural history and conservation of this stream species and to reconstruct the biodiversity history of this endemic region.

The objective of this study is to clarify the population structure of *P. ichikawai* using multiple types of abundant and high‐resolution genetic data. We investigated (1) whether current catfish populations scattered in the middle reaches of rivers retain genetic signatures shaped by paleo‐river systems formed during past marine regression periods, and (2) whether their population structure differs from that of floodplain‐dwelling freshwater fishes. To this end, we inferred the population structure of *P. ichikawai* from whole mitochondrial genome sequences (mitogenomes; ~16 kb), microsatellite polymorphisms (10 loci), and reduced‐representation genome sequences obtained by MIG‐seq [yielding more than a thousand single nucleotide polymorphisms (SNPs)], using samples from all major populations throughout the species' range. We further inferred the population formation processes of this species under Quaternary climate changes by reconstructing historical demography of selected populations based on a newly established chromosome‐level genome assembly and whole‐genome resequencing data. Using different types of genetic markers, this study documents how even weak population structures formed in the recent past can be resolved, and particularly highlights the effectiveness of genome‐wide SNP data.

## 2 Methods

### 2.1 Ethics Statement

*Pseudobagrus ichikawai* is a legally protected species that has been designated as a Natural Monument of Japan since 1977. We obtained sampling permission for this research from the Agency for Cultural Affairs, Japan. We used wild fish tissues (fin clips) collected by non‐invasive sampling. After a minimal portion of fin tissue had been collected under anesthesia with 2‐phenoxy‐ethanol, the fish were gently returned to the river or kept for the *ex situ* conservation program.

### 2.2 De Novo Whole‐Genome Sequencing

De novo whole‐genome sequencing of *P. ichikawai* was conducted on a male individual housed at the Lake Biwa Museum in Shiga Prefecture, Japan, immediately after its death (sample ID: Inabe‐02_010; Table S1). This individual was originally derived from the Inabe River system in 2011. Total DNA was extracted from the muscle tissue using the NucleoBond HMW DNA Kit (MACHEREY‐NAGEL, Germany). To obtain HiFi reads (Wenger et al. 2019), SMRTbell library preparation and HiFi sequencing on the PacBio Sequel II system were outsourced to BGI Japan.

To obtain Hi‐C reads for use in scaffolding, the Hi‐C library was constructed based on the “iconHi‐C protocol” by Kadota et al. (2020), using muscle tissue from the same individual used for the HiFi sequencing. The DpnII enzyme was employed to digest the cross‐linked DNA, and the protocol was slightly modified in the adapter ligation and PCR enrichment procedures, which were instead performed using the NEBNext Ultra II DNA Library Prep Kit for Illumina (New England Biolabs, USA) at half the recommended volume, as per the manufacturer's instructions. The constructed library was sequenced with 150‐bp paired‐end reads on the Illumina HiSeq X Ten platform at Macrogen Japan.

Acquired HiFi reads were assembled with Hifiasm v0.16.1 (Cheng et al. 2021) in the Hi‐C integrated assembly mode, using Hi‐C reads adapter‐trimmed with fastp v0.23.4 (Chen et al. 2018). Assembled contigs were scaffolded using 3D‐DNA v180922 (Dudchenko et al. 2017) based on the frequency of chimeric mapped reads resulting from the Juicer v1.6 pipeline (Durand, Shamim, et al. 2016) with default parameters. The run‐asm‐pipeline.sh script of 3D‐DNA was used to scaffold contigs with the options ‘‐i 15000 ‐r 2 ‐‐editor‐repeat‐coverage 5’ and to generate a contact map. Juicebox v2.16.00 (Durand, Robinson, et al. 2016) was used to check and manually correct assembly errors. The basic statistics (i.e., number of contigs, total length, N50, L50, and GC content) of the scaffolded genome were calculated for contigs > 1000 bp using QUAST v5.2.0 (Mikheenko et al. 2023). The integrity of the genome was assessed with BUSCO v5.4.3 (Manni et al. 2021) using 3640 actinopterygian core genes (‐l actinopterygii_odb10). To assess the levels of contamination, potential contaminants were identified and visualized as a BlobPlot using BlobTools v1.1 (Laetsch and Blaxter 2017). This scaffolded genome (AP044115–AP044587) was used as the reference genome in the following analyses.

### 2.3 Sampling and DNA Extraction

Fin clip sampling was conducted on a total of 535 individuals from 17 main streams and tributaries of 10 river systems, covering the entire range of *P. ichikawai* , collected from 1996 to 2009 (Figure 1; Tables 1 and S1). As an indicator of habitat size in each river, the length of the river course was classified into four levels (the range along the river course: (1) ≤ 100 m; (2) < 1 km; (3) < 10 km; (4) ≥ 10 km) based on the distribution survey at the time of sampling. For all individuals, a portion of the pelvic or adipose fin was excised using a minimally invasive procedure and preserved in 99% ethanol. Total DNA was extracted from the fin clips using either the standard phenol–chloroform extraction method or a commercial extraction kit, including the DNeasy Blood and Tissue Kit (Qiagen, Germany), GenElute Mammalian Genomic DNA Miniprep Kit (Sigma‐Aldrich, USA), and the Genomic DNA Purification Kit (Promega, USA).

**TABLE 1:** Sampling localities and number of specimens of *Pseudobagrus ichikawai* for each analysis.

Population	River code	River, locality	Sampling year(s)	Number of specimens			
				Mitogenome	Microsatellites	MIG‐seq	Whole genome
1	Miya‐01	Miya River, Mie Prefecture	1999, 2005	3	27	7	—
2	Miya‐02	Tributary a, Miya R. system, Mie Pref.	1997, 2001	3	36	8	—
3	Miya‐03	Tributary b, Miya R. s., Mie Pref.	2001	3	5	3	—
4	Miya‐04	Tributary c, Miya R. s., Mie Pref.	2001, 2002	8	39	8	8
5	Kumozu	Tributary of Kumozu R. s., Mie Pref.	2002, 2005	3	46	8	—
6	Suzuka	Tributary of Suzuka R. s., Mie Pref.	2008	3	35	8	—
7	Inabe‐01	Tributary a of Inabe R. s., Mie Pref.	1997, 2004, 2006	8	33	7	8
8	Inabe‐02	Tributary b of Inabe R. s., Mie Pref.	2009	3	9	8	—
9	Ibi	Tributary of Ibi R. s., Gifu Pref.	2005	3	44	5	—
10	Nagara	Tributary of Nagara R. s., Gifu Pref.	1996	8	120	24	8
11	Kiso‐01	Tributary a, Kiso R. s., Gifu Pref.	2001	2	14	3	—
12	Kiso‐02	Tributary b, Kiso R. s., Gifu Pref.	1997	3	12	8	—
13	Shonai	Tributary of Shonai R. s., Gifu Pref.	1997	2	3	3	—
14	Yahagi	Tributary of Yahagi R. s., Aichi Pref.	1997	3	28	8	—
15	Toyo‐01	Toyo R., Aichi Pref.	1996	11	33	8	11
16	Toyo‐02	Tributary a, Toyo R. s., Aichi Pref.	1997	2	16	8	—
17	Toyo‐03	Tributary b, Toyo R. s., Aichi Pref.	1996	3	35	7	—
	Total			71	535	131	35

### 2.4 Microsatellite Analysis

A total of 535 specimens from the 17 sampling sites were analyzed using 10 microsatellite loci isolated from *P. ichikawai* (see Watanabe et al. 2001), including eight loci with dinucleotide repeats (Pi‐h03, Pi‐h19, Pi‐h33, Pi‐h34, Pi‐h39, Pi‐h42, Pi‐h61, and Pi‐m41) and two with tetranucleotide repeats (Pi‐m59 and Pi‐m105). PCR conditions for each locus were described by Watanabe et al. (2001). PCR products were sized on an automated DNA sequencer (ABI Prism GA310; Applied Biosystems, USA) using GeneScan v3.1 and ROX400HD as the size standard (Applied Biosystems). No missing data were included in this data set. The resulting data set is referred to as the “microsatellite data set.”

As genetic diversity and contemporary population size indices, expected heterozygosity (*H*_e_) and effective population size (*N*_e_) were calculated. The *H*_e_ was calculated using Arlequin v3.5 (Excoffier and Lischer 2010). The *N*_e_ was estimated using NeEstimator v2.1 (Do et al. 2014) with the linkage disequilibrium method. To elucidate the population structure, we performed principal component analysis (PCA), uniform manifold approximation and projection (UMAP; McInnes et al. 2020), *F*_ST_ calculation, clustering analysis, and phylogenetic inference. PCA was conducted based on the correlation matrix of allele frequencies among individuals using GenoDive v2.0b27 software (Meirmans and Van Tienderen 2004). UMAP was applied to all 10 principal components using the umap function implemented in the R package umap v0.2.10.0 (McInnes et al. 2020). Population pairwise *F*_ST_ values were calculated using Arlequin. The statistical significance of *F*_ST_ was tested with 1000 permutations in Arlequin and adjusted using Holm's correction for multiple comparisons, with statistical significance determined at a corrected *p* < 0.05. Unsupervised clustering was performed using PopCluster v1.1 (Wang 2022) at each number of genetic populations (*K*) from 1 to 17, and the following settings were used: medium scaling, 100 replicated runs for each *K* value, admixture analysis model, relatedness calculation by the estimator of Wang (2002), and allele frequency prior determined by the program. The best *K* was chosen based on two estimators, *D*_LK2_ and *F*_STIS_ (Wang 2022). For phylogenetic inference, a neighbor‐joining tree based on the (*δμ*)^2^ distance (Goldstein et al. 1995) was constructed using POPTREEW (Takezaki et al. 2014).

### 2.5 MIG‐Seq Analysis

The multiplexed inter‐simple sequence repeat (ISSR) genotyping‐by‐sequencing (MIG‐seq) method (Suyama and Matsuki 2015) was applied to 152 specimens selected from the same 17 sampling sites used in the microsatellite analysis (Table 1). We selected these specimens to maximize the efficiency of NGS‐based data acquisition while ensuring a comprehensive population genetic analysis across all sampling sites. After initial SNP calling, 131 specimens were retained for analysis, selecting only those with a missing rate below 10% per individual (see below). MIG‐seq library preparation followed the original protocol (Suyama and Matsuki 2015), with some modifications, including changing the sequencing platform from MiSeq to NovaSeq (Illumina, USA) to enhance data volume and cost efficiency using modified primer sets (Onuki and Fuke 2022; Table S2). The first round of PCR (1st PCR) used eight ISSR primer sets at 38°C, with the Multiplex PCR Assay Kit v.2 (Takara, Japan). Diluted 1st PCR products were then used for the second round of PCR (2nd PCR), which incorporated barcoded primers for individual identification (Onuki and Fuke 2022). The 2nd PCR was performed using Phusion High‐Fidelity PCR Master Mix with HF Buffer (New England Biolabs, USA), running 20 cycles at 98°C for 30 s, 54°C for 15 s, and 68°C for 30 s. The products were pooled, purified, and size‐selected (300–800 bp) using the MinElute PCR Purification Kit (Qiagen, Germany), SPRIselect (Beckman Coulter, USA), and GeneRead Size Selection Kit (Qiagen, Germany). TapeStation (Agilent Technologies, USA) was used to confirm fragment size and estimate final concentration. The final libraries were sequenced on an Illumina NovaSeq platform (150‐bp paired‐end mode) at Novogene (Beijing, China). Sufficient data could not be obtained for 10 specimens; thus, fastq data from the remaining 142 individuals were used in the subsequent analysis.

For all acquired data, adapter trimming and read quality control were performed using fastp. Reads with > 40% unqualified bases (i.e., bases with a quality score < 30; ‐q 30) were excluded (‐u 40). These reads were mapped to the reference genome generated in this study using bwa‐mem2 v2.2 (Vasimuddin et al. 2019). Alignments in SAM format were sorted and compressed into BAM format using Samtools v1.16.1 (Danecek et al. 2021). All multi‐mapped reads were excluded (grep ‐v ‐e ‘XA:Z:’ ‐e ‘SA:Z:’). SNP calling was performed using the ref_map.pl pipeline in Stacks v2.62 (Rochette et al. 2019). When calling SNPs by populations in Stacks, loci common to more than 90% of all samples (*R* = 0.9) were retained. Sites with less than two minor alleles (‐‐min‐mac = 2) were excluded, and the ‐‐ordered‐export option was used according to the software's recommendation for reference‐aligned data. All other parameters were left at their default settings. SNPs were initially called using all 142 individuals and then again using 131 individuals, excluding the 11 individuals with a missing rate per individual > 10% (Table S1). The *π* and *H*_e_ values for MIG‐seq data were calculated using the populations program in Stacks. We pruned SNPs in linkage disequilibrium using Plink v1.9 (Chang et al. 2015) (www.cog‐genomics.org/plink/1.9/) with the ‐‐indep‐pairwise 50 10 0.1 option, which removes one SNP from each pair with *r* ^2^ > 0.1 in a sliding window of 50 SNPs, shifted by 10 SNPs at each step. This resulting data set is referred to as the “MIG‐seq data set.”

To elucidate the population structure, we performed PCA, UMAP, *F*_ST_ calculation, clustering analysis, and phylogenetic inference using the MIG‐seq data set, largely following the procedures used for the microsatellite analysis, with some modifications reflecting intrinsic differences between microsatellite and SNP data. Specifically, PCA was conducted using a variance‐standardized relationship matrix implemented in Plink instead of allele‐frequency‐based PCA in GenoDive, the number of principal components used for UMAP was adjusted (top 20 PCs) to adequately capture genome‐wide SNP variation, and phylogenetic inference was performed using a neighbor‐net algorithm based on Hamming distance implemented in SplitsTree v4.18.2 (Huson and Bryant 2006) with 1000 bootstrap runs.

Additionally, to assess the probability of admixture after population divergence, we conducted *f*_3_ tests (Patterson et al. 2012), which evaluate the correlation of allele frequencies across genome‐wide markers. The tests were performed for all 2040 population triplets derived from the 17 populations (i.e., all 136 population pairs × all 15 possible outgroups) using ADMIXTOOLS 2 v2.0 (Maier et al. 2023). The *f*_3_ tests with *Z*‐scores less than −3.0 were considered significant.

### 2.6 Whole‐Genome Resequencing Analysis

To obtain mitogenome sequences and reconstruct population demography, whole‐genome resequencing was performed on 71 specimens selected from the same 17 sampling sites (Table 1; Table S1). We selected these specimens to obtain mitogenome sequences across all sites, while focusing on four geographically distant populations to conduct historical demographic analyses. Libraries were prepared using the NEBNext Ultra II FS DNA Library Prep Kit for Illumina (New England Biolabs, USA). Indexing was performed using NEBNext Multiplex Oligos for Illumina (New England Biolabs, USA). Sequencing was performed using a HiSeq X Ten (Illumina, USA) or DNBSEQ‐G400 (MGI, China) in 150‐bp paired‐end mode. Sequencing was outsourced to Macrogen Japan or BGI Japan. For all acquired data, read quality control was performed using fastp. Reads with > 40% unqualified bases (i.e., bases with a quality score < 20; ‐q 20) were excluded (‐u 40). A four‐base sliding window was applied from both the 3′ and 5′ ends of the reads, and windows with a mean quality score < 20 were removed (–3 –5). Among all samples, 36 were sequenced at approximately 4× coverage for mitogenome assembling. The remaining 35 samples were sequenced at approximately 20× coverage for genetic diversity estimation and demographic analyses, as well as mitogenome assembly (Table 1).

For the filtered reads from the 71 specimens, de novo mitogenome assembly was performed using GetOrganelle v1.7.5 (Jin et al. 2020). Short‐read sequencing data consisted of 5–15 million reads per individual. The animal_mt option was used. All runs employed k‐mer sizes of 21, 55, 85, and 115. This resulted in complete mitogenome sequences for all individuals (approximately 16,530 bp). All sequences were annotated using MitoAnnotator v3.85 (Iwasaki et al. 2013).

For the 35 samples from four selected populations sequenced at approximately 20× coverage, SNP calling was conducted as follows. The filtered reads were mapped to the reference genome using bwa‐mem2. Alignments in SAM format were sorted and compressed into BAM format as in the MIG‐seq analysis. PCR duplicates created during library preparation were detected using MarkDuplicates in Picard v2.25.0 (http://broadinstitute.github.io/picard). Two types of SNP calls were performed. First, SNPs and invariant sites were called using mpileup in bcftools v1.16 (Danecek et al. 2021; Li 2011). In addition, sites with excessively low or high depth and indels were filtered out using vcftools v0.1.16 (Danecek et al. 2011) under the following conditions: ‐‐remove‐indels, ‐‐max‐meanDP = 28.5 (twice the mean depth from bcftools), ‐‐minDP = 8. Sites with missing data were also filtered using vcftools with ‐‐max‐missing = 0.8. The resulting data set is referred to as the “all‐sites data set.” Second, for pairwise sequentially Markovian coalescent (PSMC) and multiple sequentially Markovian coalescent (MSMC) analyses, SNPs were called using bcftools and bamCaller.py in msmc‐tools (https://github.com/stschiff/msmc‐tools). After sites on chromosomal scaffolds were selected, those with a minimum mapping quality < 20 (‐‐min‐MQ 20), a minimum base quality < 20 (‐‐min‐BQ 20), or depth greater than twice the chromosome‐wide average per individual were filtered out. The resulting data set is referred to as the “PSMC data set.”

To assess the genetic diversity within each *P. ichikawai* population, we estimated *π* value and runs of homozygosity (ROH) using the all‐sites data set. *π* values were calculated in non‐overlapping 10 kb windows using pixy v1.2.7 (Korunes and Samuk 2021). ROH lengths were estimated, and inbreeding coefficients (*F*_ROH_) were derived as the proportion of the genome covered by ROHs of different lengths (≥ 100 kb and ≥ 1 Mb). This analysis was performed using the roh command in bcftools with the options ‐‐AF‐dflt 0.4 and ‐G 30.

Historical demography of the four selected populations (each including 8–11 individuals) was estimated using the PSMC (Li and Durbin 2011), MSMC (Schiffels and Durbin 2014), SMC++ (Terhorst et al. 2017), and PopSizeABC methods (Boitard et al. 2016). These methods were selected because, empirically, they offer different windows of resolution across time: PSMC, hundreds to hundreds of thousands of generations ago; MSMC, one hundred to hundreds of thousands; SMC++, tens to one hundred thousand; and PopSizeABC, several to hundreds of thousands, although the exact range depends on the data and true demographic history (Nadachowska‐Brzyska et al. 2022). We integrated these approaches to comprehensively reconstruct historical demography from the deep past to the near present.

PSMC and MSMC analyses were performed using msmc v2.1.3 (Schiffels and Wang 2020). PSMC was performed as PSMC' using PSMC data set with default settings for each individual. MSMC was performed using PSMC data set phased with SHAPEIT2 (Delaneau et al. 2012). VCF files for each individual in the PSMC data set were merged (bcftools merge ‐‐missing‐to‐ref) and phased with read‐aware phasing (Delaneau et al. 2013) using SHAPEIT2, after removing multiallelic sites. The phased VCFs were then split by individual (bcftools split), and sites containing only the reference alleles were removed. For each population, MSMC was run using the phased VCFs with default settings, except that sites with ambiguous phasing were skipped (‐s). The relative cross‐coalescence rate (rCCR) was simultaneously calculated following Schiffels and Wang (2020) to estimate divergence time of each population pairs. For MSMC and rCCR calculations, 30 bootstrap runs were conducted using multihetsep_bootstrap.py from msmc‐tools. The chunk size, number of chunks per chromosome, and number of chromosomes were set to 1 Mbp, 25, and 28, respectively. For both PSMC and MSMC, regions with low mappability were masked with a (150, 2)‐mappability BED file generated using GenMap v1.3.0 (Pockrandt et al. 2020). The generation time and mutation rate per site per generation were assumed to be 2 years (Watanabe 1994a, 2008) and 3.50 × 10^−9^ (Malinsky et al. 2018), respectively.

SMC++ analysis was performed with the merged PSMC data set. Unlike MSMC, SMC++ does not require phased data and is thus robust to phasing errors (Terhorst et al. 2017). For each population, one representative individual with the highest coverage was designated as the distinguished lineage (Miya‐04_07, Inabe‐01_08, Nagara_20, Toyo‐01_09). Regions of low mappability were masked based on the (150, 2)‐mappability calculated above. The number of spline knots was set to 20, while other parameters were left at their default settings. Divergence time under the “clean split” model between each population pair was also estimated. Twenty bootstrap runs were performed using bootstrap_smcpp.py (https://github.com/biozzq/goat‐population‐genetic/blob/master/04.demographic/bootstrap_smcpp.py). The same chunk size, number of chunks per chromosome, and number of chromosomes as used in the MSMC bootstrap were applied. The generation time and mutation rate per site per generation were assumed to be the same as in the other analyses.

More recent historical demography was estimated using PopSizeABC (Boitard et al. 2016) with the merged PSMC data set. Two summary statistics, allele frequency spectrum and linkage disequilibrium, were used in the approximate Bayesian computation (ABC) analysis. These statistics were calculated for 21 discrete time windows (with the oldest window starting 30,000 generations ago) from the empirical dataset and compared with statistics from simulated data sets. The minor allele frequency threshold for calculating linkage disequilibrium was set to 0.2 to minimize prediction error. Linkage disequilibrium was summarized in segments of 2,000,000 bp. For the simulated datasets, the number of segments was set to 30 (each 2,000,000 bp in size), and the number of simulated data sets was set to 1000,000. Log‐uniform priors were applied for effective population size (*N*_e_) ranging from 10 to 500,000 (log_10_‐scale: 1–5.7), and for recombination rate ranging from 0.1 × 10^−8^ to 1 × 10^−8^. Generation time and mutation rate per site per generation were assumed to be the same as in the other analyses (2 years and 3.50 × 10^−9^). ABC estimates of population sizes were obtained using neural network regression with a tolerance of 0.001.

## 3 Results

### 3.1 De Novo Whole‐Genome Sequencing, MIG‐Seq, and Resequencing

To provide a genomic basis for our study, we first established a high‐quality, chromosome‐level reference genome for *Pseudobagrus ichikawai* . De novo whole‐genome sequencing yielded a chromosome‐level assembly of the *P. ichikawai* genome. Genome assembly and scaffolding using 1,307,375 HiFi reads and 372,814,414 Hi‐C reads produced a genome sequence of 675 Mb, consisting of 473 contigs with a contig N50 of 23.5 Mb and an L50 of 13. The number of contigs > 5 Mb was 28, matching the chromosome number (*n*) of this species (Ueno 1985; Figure S1). The BUSCO scores were 92.2%, 2.8%, and 0.7% for single‐copy, duplicated, and fragmented genes, respectively, with 4.9% missing. Contamination in the scaffolded assembly from microbiomes or other organisms was negligible (Figure S2). The present assembly shows significant improvements in both contiguity and completeness compared to the draft genome assembled in a previous study (Mizuno et al. 2025), which consisted of 65,543 scaffolds with an N50 of 0.3 Mb and BUSCO scores of 74.7% and 1.1% for single‐copy and duplicated genes, respectively, reflecting the improved resolution provided by HiFi and Hi‐C technologies.

Using the obtained assembly as a reference genome, we performed MIG‐seq and resequencing analyses to generate sufficient genome‐wide SNPs. MIG‐seq analysis of 131 specimens yielded an average of 0.301 M reads per individual (s.d. 0.071 M reads), with an average coverage of 13.02× (s.d. 4.03×) (Table S3). The resulting MIG‐seq data set contained 1259 SNPs. Low‐coverage whole‐genome resequencing for 36 specimens yielded an average of 10.15 M reads per individual (s.d. 2.65 M reads) (Table S3). High‐coverage whole‐genome resequencing for 35 specimens yielded an average of 114.45 M reads per individual (s.d. 11.99 M reads), with an average coverage of 17.20× (s.d. 2.02×) (Table S3). The number of sites in each data set was as follows: all‐sites dataset, 512,558,842 sites with 1,115,097 SNPs; PSMC data set, 178,450 SNPs on average (Table S3).

### 3.2 Genetic Diversity

We assessed the levels of genetic diversity within *P. ichikawai* populations by comparing estimates derived from mitogenomes, microsatellites, and genome‐wide SNPs. Genetic diversity in *P. ichikawai* was consistently low across different markers, and estimates from these markers were generally correlated. A total of 34 mitogenome haplotypes (16,526–16,529 bp) were detected from 71 specimens (2–11 specimens per population) collected from 17 rivers in 10 river systems (Figure 1; Tables 1 and S1). Their sequence differences were small, with 1–16 segregating sites between pairs (0.006%–0.097%) and 72 segregating sites in the full alignment (0.436%). The first half of the control region was completely monomorphic, consistent with a previous study (i.e., monomorphic in 420 bp for 75 specimens from eight river systems; Watanabe and Nishida 2003). Insertions and deletions, observed only in the 16S rRNA, tRNA, and control regions, totaled eight sites. Among the 47 substitutions in the coding regions, 20 were nonsynonymous, including 17 in NADH subunits [ND 1 (3), 2 (1), 4 L (1), 4 (5), 5 (6), and 6 (1)], two in cytochrome *b*, and one in cytochrome *c* oxidase subunit 2 regions. Each population sample possessed 1–5 mitogenome haplotypes, with haplotype diversity (*h*) of 0–1.000 (average ± SD = 0.729 ± 0.360) and nucleotide diversity (*π*) of 0–0.000605 (0.000212 ± 0.000198) (Table 2). The populations from the Miya River system (populations 1.Miya‐01, 2.Miya‐02, and 3.Miya‐03) showed the highest diversity (*h*: 0.857–1.000, *π*: 0.000132–0.000605).

**TABLE 2:** Comparison of population genetic diversity of *Pseudobagrus ichikawai* across different genetic markers to evaluate the correlation and consistency among mitochondrial, microsatellite, and SNP‐based estimates.

Population	River code	Range	Mitogenome			Microsatellites (10 loci)			MIG‐seq (2607 SNPs)			Reseq (1,115,097 SNPs)		
			*n*	*h*	*π*	*n*	*H*_e_	*N*_e_	*n*	*H*_e_	*π*	*n*	*π*	*F*_ROH_
1	Miya‐01	4	3	1.000	0.000484	27	0.664 ± 0.185	17 (12–26)	7	0.00009 ± 0.00001	0.00010	—	—	—
2	Miya‐02	4	3	1.000	0.000403	36	0.546 ± 0.199	171 (45–inf.)	8	0.00008 ± 0.00001	0.00009	—	—	—
3	Miya‐03	3	3	1.000	0.000605	5	0.478 ± 0.241	n.a.	3	0.00007 ± 0.00001	0.00009	—	—	—
4	Miya‐04	3	8	0.857	0.000132	39	0.679 ± 0.095	16 (12–23)	8	0.00010 ± 0.00001	0.00011	8	0.00028	0.130–0.271
5	Kumozu	3	3	0.000	0.000000	46	0.437 ± 0.253	27 (15–61)	8	0.00010 ± 0.00001	0.00010	—	—	—
6	Suzuka	2	3	0.000	0.000000	35	0.108 ± 0.162	13 (1–inf.)	8	0.00007 ± 0.00000	0.00008	—	—	—
7	Inabe‐01	1	8	0.429	0.000052	33	0.391 ± 0.265	4 (3–10)	7	0.00009 ± 0.00001	0.00010	—	0.00020	0.172–0.254
8	Inabe‐02	1	3	0.667	0.000040	9	0.318 ± 0.272	n.a.	8	0.00009 ± 0.00001	0.00009	—	—	—
9	Ibi	3	3	1.000	0.000403	44	0.521 ± 0.203	27 (14–70)	5	0.00008 ± 0.00001	0.00009	—	—	—
10	Nagara	3	8	0.429	0.000026	120	0.688 ± 0.114	43 (35–54)	24	0.00012 ± 0.00001	0.00012	8	0.00030	0.372–0.493
11	Kiso‐01	4	2	1.000	0.000060	14	0.566 ± 0.133	27 (7–inf.)	3	0.00009 ± 0.00001	0.00011	—	—	—
12	Kiso‐02	3	3	0.667	0.000161	12	0.368 ± 0.216	8 (2–inf.)	8	0.00010 ± 0.00001	0.00011	—	—	—
13	Shonai	3	2	1.000	0.000121	3	0.527 ± 0.292	n.a.	3	0.00010 ± 0.00001	0.00012	—	—	—
14	Yahagi	3	3	1.000	0.000323	28	0.674 ± 0.113	32 (18–84)	8	0.00013 ± 0.00001	0.00014	—	—	—
15	Toyo‐01	4	11	0.346	0.000022	33	0.334 ± 0.311	24 (9–196)	8	0.00007 ± 0.00000	0.00008	11	0.00022	0.432–0.572
16	Toyo‐02	2	2	1.000	0.000363	16	0.456 ± 0.273	27 (7–inf.)	8	0.00010 ± 0.00001	0.00011	—	—	—
17	Toyo‐03	3	3	1.000	0.000403	35	0.710 ± 0.157	64 (37–163)	7	0.00014 ± 0.00001	0.00015	—	—	—

*Note:* Range indicates the length of the river course as an indicator of habitat size (1, ≤ 100 m; 2, < 1 km; 3, < 10 km; 4, ≥ 10 km). *n* denotes the number of individuals used. *h* represents haplotype diversity, and *π* indicates nucleotide diversity. *H*_e_ is the expected heterozygosity estimated from microsatellite or SNP data. *N*_e_ is the effective population size estimated using the linkage disequilibrium method with a lowest allele frequency of 0.05; 95% confidence intervals are shown in parentheses, with values labeled as “n.a.” not calculated due to small sample sizes and “inf.” indicating infinity. *F*_ROH_ represents the proportion of runs of homozygosity (≥ 100 kb) relative to the whole genome.

Expected heterozygosity (*H*_e_) for microsatellites (10 loci) ranged from 0.108 to 0.710 (0.158 ± 0.498), and for MIG‐seq data (1259 SNPs), from 0.00007 to 0.00014 (0.00010 ± 0.00002) (Table 2). Population 17.Toyo‐03 showed the highest heterozygosity and population 6.Suzuka showed the lowest heterozygosity for both markers. Population genetic diversity (*H*_e_) for microsatellites and MIG‐seq was positively correlated (Kendall's *τ* = 0.478, *p* = 0.011 for *H*_e_; *τ* = 0.558, *p* < 0.01 for *π*) (Figure S3). The genetic diversities for mitogenomes also showed positive correlation with those for microsatellites (*τ* = 0.392, *p* = 0.043 for *h* and *H*_e_) but not with those of MIG‐seq (*τ* < 0.30, *p* > 0.1) (Figure S3). No correlation was detected between all of these genetic diversity indices and the index of distribution range in the river course, as a proxy for habitat size (*τ* < 0.33, *p* > 0.1).

The effective population size (*N*_e_) estimated by the linkage disequilibrium method using microsatellite data ranged from 4 (7.Inabe‐01) to 171 (2.Miya‐02) (Table 2), although the confidence intervals of *N*_e_ were generally large, making it difficult to estimate *N*_e_ for some populations with small sample sizes. *N*_e_ could not be estimated from the MIG‐seq data for the same reason. The *N*_e_ was positively correlated with *H*_e_ of microsatellites (*τ* = 0.443, *p* = 0.031). On the other hand, no correlation was detected between *N*_e_ and the genetic diversity indices (*H*_e_, *π*) from MIG‐seq data (*τ* < 0.31, *p* > 0.1). Similarly, no correlation was found between *N*_e_ and the index of distribution range in the river course (*τ* < 0.31, *p* > 0.1) (Figure S3).

The genome‐wide average *π* value, calculated in non‐overlapping 100 kb windows for the four re‐sequenced populations (populations 4, 7, 10, and 15), ranged from 0.00020 to 0.00030. These values showed a pattern similar to those observed for microsatellite‐based *π* and heterozygosity from MIG‐seq, with higher values in populations 4.Miya‐04 and 10.Nagara, and lower values in 7.Inabe‐01 and 15.Toyo‐01 (Table 2). Consistently, the proportion of ≥ 100 kb ROHs in the whole genome (*F*_ROH_), an indicator of inbreeding, ranged from 0.130 to 0.572; lower in 4.Miya‐04 and 10.Nagara, and higher in 7.Inabe‐01 and 15.Toyo‐01 (Table 2; Figure S4). The *F*_ROH_ based on ROHs ≥ 1 Mb was particularly high in 7.Inabe‐01 (0.0127–0.0795), suggesting a recent bottleneck and elevated inbreeding in this population.

### 3.3 Population Structure

**FIGURE 2:**
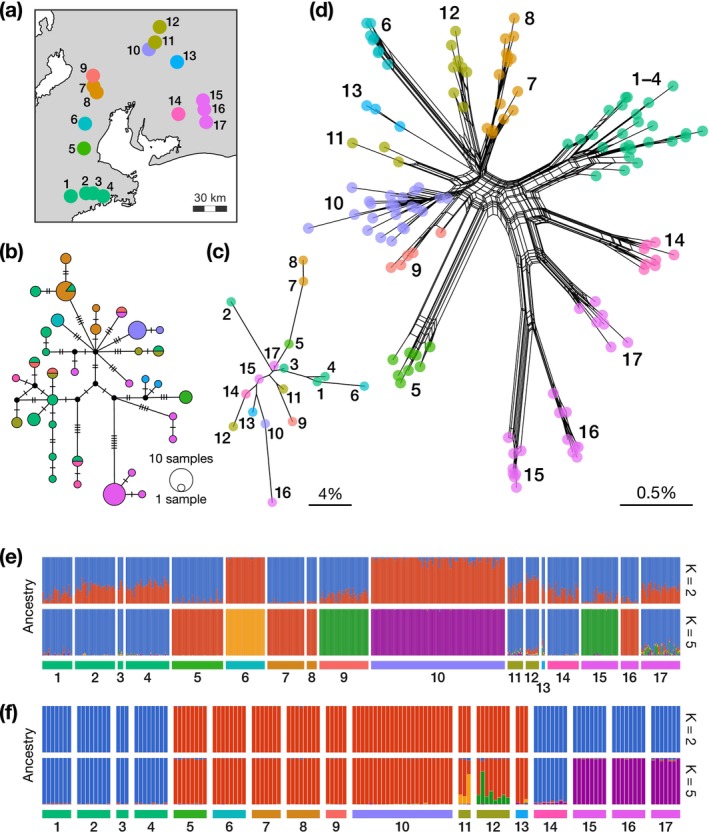
Inferred population structure of *Pseudobagrus ichikawai* using three genetic markers: Complete mitogenomes (approximately 16,530 bp), microsatellites (10 loci), and genome‐wide SNPs (1259 SNPs). The bold numbers indicate the sampling sites, and the colors indicate the river systems, corresponding to those in Figure 1. (a) Location of sampling sites and river systems. (b) Haplotype network based on complete mitogenome data. (c) Neighbor‐joining tree based on the (*δμ*)^2^ distance using microsatellite data. (d) Neighbor‐net based on the Hamming distance using genome‐wide SNPs. (e, f) Results of unsupervised clustering using microsatellites and genome‐wide SNPs. Estimated individual admixture proportions with PopCluster at *K* = 2 and 5 using (e) microsatellite data and (f) genome‐wide SNPs.

In characterizing the spatial organization of genetic variation, we compared population structures inferred from different marker types, including mitochondrial DNA, microsatellites, and MIG‐seq data. Significant genetic differentiation was detected among almost all *P. ichikawai* populations, based on population pairwise *F*_ST_ values calculated from both microsatellite and MIG‐seq data with a moderately large sample size (≥ 7 individuals) (Table S4). In contrast, the complete mitogenome sequences, which exhibited slight diversity, showed no clear geographic structure in their haplotype network (Figure 2b). Specifically, haplotypes from the same river systems were dispersed across the network, and some were frequently shared among geographically distant populations. This pattern likely reflects incomplete lineage sorting.

Microsatellite data revealed a weak geographic signal. Although the population network based on the microsatellite (*δμ*)^2^ distances showed some clustering by river system, populations from different tributaries within the same system were often scattered (Figure 2c). This pattern was also supported by barplots of the PopCluster analysis with the best *K* of 2 and 5 (based on *D*_LK2_ and *F*_STIS_ estimators, respectively) and its surrounding *K*s; the tributary samples clustered together in some river systems, such as the Miya River (populations 1–4) and the Inabe River (populations 7 and 8), but others clustered with samples from different river systems or clustered separately (Figures 2e, S5a). PCA and UMAP plots also showed similar trends (Figure S6a,c).

In contrast, the MIG‐seq data demonstrated clear clustering of individual specimens by population, with geographic population structure well represented in the neighbor‐net and barplots of PopCluster (Figure 2d,f). The populations were primarily divided into two groups: the northern group (populations 5–13) and the eastern + western group (1–4, Miya River; 14, Yahagi River; 15–17, Toyo River), with the latter indicating the trans‐Ise Bay distribution pattern. The latter group was further separated at *K* ≥ 4 in the PopCluster barplots, with the three Toyo River populations (15–17) and others being separated (Figures 2f, S5b). The neighbor‐net showed substantial divergence among the three tributary populations within the Toyo River system (populations 15–17), comparable to their divergence from the adjacent Yahagi River population (14; Figure 2d). Also, the 5.Kumozu population showed differentiation from other populations of the northern group. PCA and UMAP plots supported these patterns (Figure S6b,d). The *f*_3_ tests detected no population triplets with statistically significant negative *f*_3_ statistics (the smallest *Z*‐score was −1.08; Table S5), suggesting no admixture among populations after their differentiation.

### 3.4 Historical Demography

**FIGURE 3:**
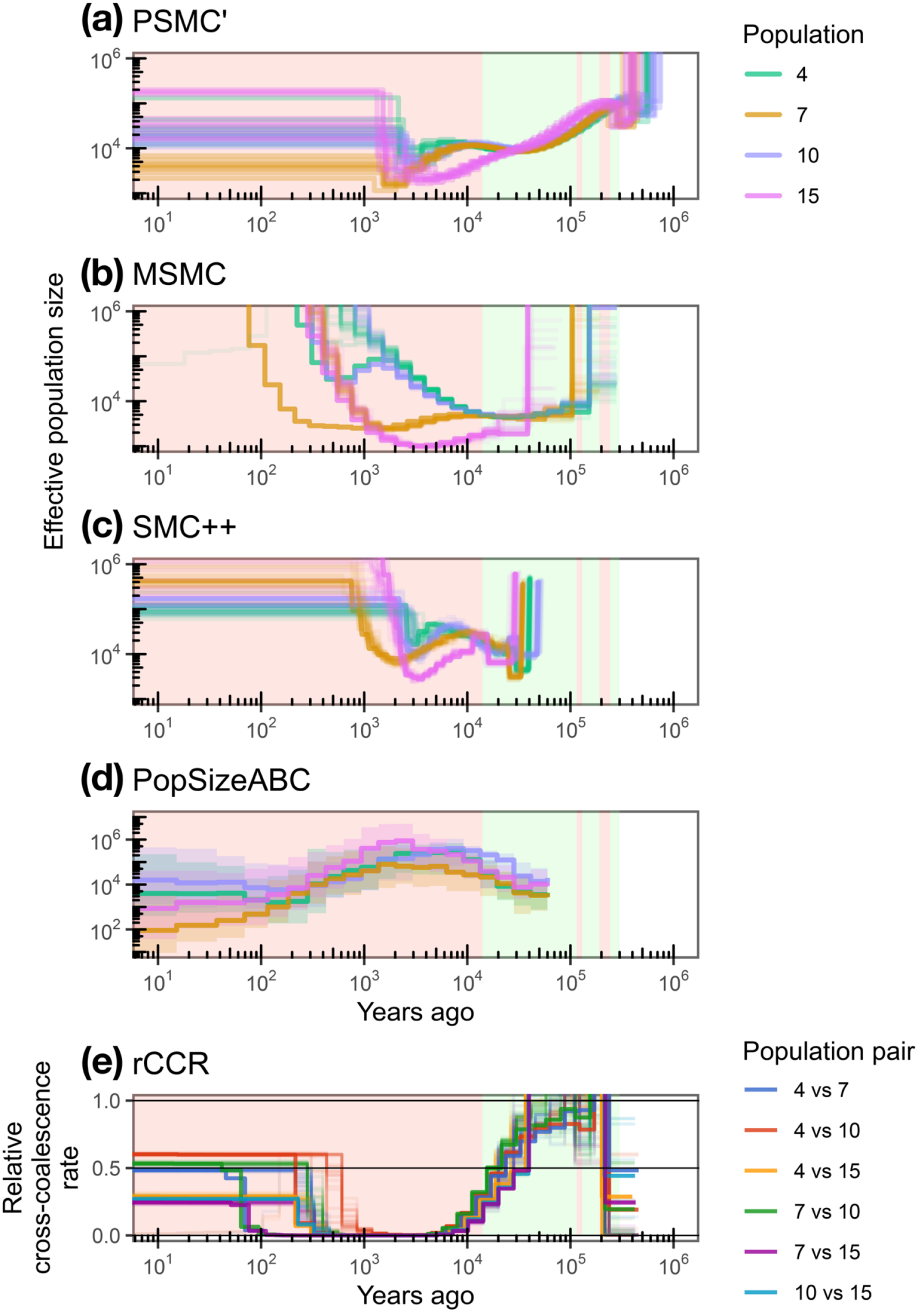
Estimated historical demography and relative cross‐coalescent rate of *Pseudobagrus ichikawai* using whole‐genome resequencing data. Historical demography was inferred with (a) PSMC', (b) MSMC, (c) SMC++, and (d) PopSizeABC methods. (e) Relative cross‐coalescent rate (rCCR) was inferred using the MSMC method. In the MSMC, SMC++, and rCCR results, the light lines represent the results of block bootstrapping runs; in the PopSizeABC results, the lines represent the median values, and the ribbons represent 95% credible intervals. In all panels, the green background indicates glacial periods, and the red background indicates interglacial or post‐glacial periods after 300 kya.

To understand the long‐term population dynamics and divergence history of *P. ichikawai* , we reconstructed the historical changes in effective population size (*N*_e_) using multiple genomic inference methods. Using the whole‐genome resequencing data for 8–11 individuals from each of the selected four population samples (populations 4, 7, 10, and 15), historical dynamics of *N*_e_ were estimated for the range of ca. 10^2^–10^5^ years ago (Figure 3). The PSMC plots for specimens of each population revealed population‐specific patterns of the dynamics for the period 10^4^–10^5^ years ago, with one of the four populations (population 15.Toyo‐01) showing a pattern different from the others. That is, after a continuous decreasing trend common to all four populations from 3 × 10^5^ to 3 × 10^4^ years ago (including the beginning of the Last Glacial Period; Lisiecki and Raymo 2005), three populations (4, 7, and 10) once experienced a stable period around 1–3 × 10^4^ years ago before rapidly decreasing, while the other population (15) showed a continuous decrease since 3 × 10^5^ years ago (Figure 3a). More recent dynamics (ca. 2 × 10^3^–3 × 10^4^ years ago), estimated by the MSMC method, showed a similar pattern of *N*_e_ between the 4.Miya‐04 and 10.Nagara populations, with an immediate increase in the post‐glacial period (Figure 3b). This pattern was also demonstrated in the SMC++ analysis (Figure 3c). Further recent dynamics (ca. 10^2^–5 × 10^3^ years ago), estimated by PopSizeABC, showed a population size reduction from 3 × 10^3^ to about 2 × 10^2^ years ago for all four populations (Figure 3d). Subsequently, stable population sizes were observed for 4.Miya‐04 and 10.Nagara, while further reductions were shown for 7.Inabe‐01 and 15.Toyo‐01. The declining trend in *N*_e_ for the latter two populations was consistent with their lower contemporary *N*_e_, lower genetic diversity, and higher *F*_ROH_ described above.

The relative cross coalescence rates (rCCR) between all population pairs in the MSMC analysis reached 0.5 around 1.5–3 × 10^4^ years ago, during the Last Glacial Period (Figure 3e), this timing being an estimate of population divergence time (Schiffels and Wang 2020). The earliest divergence occurred between 7.Inabe‐01 and 15.Toyo‐01, while the latest occurred between 7.Inabe‐01 and 10.Nagara. This order of divergence is consistent with the results of the population structure analysis and their geographic distances. The SMC++ analysis also inferred comparable divergence times (around 1.0–2.75 × 10^4^ years ago) and a similar order of divergence (Figure S7).

## 4 Discussion

This study examined population structure in *Pseudobagrus ichikawai* using multiple genetic markers with different properties. By analyzing mitogenomes, microsatellites, and genome‐wide SNPs derived from reduced‐representation and whole‐genome resequencing in largely overlapping sets of populations, we empirically compared their ability to capture genetic signals in a species characterized by extremely low genetic diversity. Although the theoretical detection limits of each marker were not explicitly evaluated, our results highlight that combining cost‐effective reduced‐representation sequencing with whole‐genome resequencing is increasingly practical and effective for capturing shallow population differentiation and demographic history under realistic conditions.

### 4.1 Low Genetic Diversity

The mitogenome and genome‐wide SNP data confirmed the low genetic diversity of *P. ichikawai* . Consistent with a previous study (Watanabe and Nishida 2003), no variation was detected in the 5′‐half of the mtDNA control region, which is typically hypervariable in vertebrates (Aquadro and Greenberg 1983; Bronstein et al. 2018). The total number of variable sites in the whole mitogenomes was 75 (0.45%), including 24 nonsynonymous substitutions, mostly in NADH subunit regions, whereas the cytochrome *c* oxidase subunit and ATPase regions predominantly had synonymous substitutions. In addition, the genome‐wide mean value of nucleotide diversity (*π*), calculated from the resequencing data, was as low as 0.00020–0.00030, representing one of the lowest levels ever reported in endangered vertebrates, e.g., mountain gorilla (0.00065), Amur tiger (0.00049), cheetah (0.00019–0.00021), and a latid fish ( *Lates japonicus* ) (0.00033–0.00034) (Cho et al. 2013; Dobrynin et al. 2015; Hashiguchi et al. 2024; Xue et al. 2015). This is also consistent with previously reported data (0.00016) from more recent samples of the Toyo River population (corresponding to our 15.Toyo‐01) (Mizuno et al. 2025). The *F*_ROH_ values calculated from the resequencing data (using ≥ 100 kb ROH) were also high, ranging from 0.13 to 0.57, likely reflecting a high degree of inbreeding (Ceballos et al. 2018), particularly in the 7.Inabe‐01 and 15.Toyo‐01 populations. The genetic diversity indices for each population showed roughly consistent patterns across microsatellite, MIG‐seq, and resequencing data, suggesting that these multilocus nuclear DNA markers can be effectively used to evaluate genetic diversity. However, these nuclear DNA indices did not correlate with the mitogenome indices, which may be due to the small sample size used for mitogenome analyses (i.e., 3 individuals per population in most cases) or stronger genetic drift resulting from smaller *N*_e_ for maternally inherited mitogenomes.

The low genetic diversity may reflect both historical and recent small effective population sizes of *P. ichikawai* . This species is restricted to the upper and middle reaches of short rivers (with total lengths ranging from a few tens to 230 km) in the Ise Bay area and hence may have experienced bottlenecks due to its limited habitat size. Although no significant relationships were observed between current habitat size and genetic diversity or *N*_e_, such bottlenecks are supported by the finding that the population size of 15.Toyo‐01, located in the upper reaches of the Toyo River, has historically remained small compared to other populations (Figure 3). The strong sedentary tendency and polygamous breeding system of this species (Watanabe 1994a, 1994b, 2008) may have resulted in a small effective population size relative to the ecological population size (i.e., small *N*_e_/*N*), leading to reduced *N*_e_. Furthermore, the populations of this species have seriously declined due to water pollution and the loss of microhabitats caused by artificial river modifications, resulting in its classification as VU on the IUCN Red List and EN on the Japan Ministry of the Environment Red List (International Union for Conservation of Nature 2022; Japan Ministry of the Environment 2020). This situation may be strongly reflected in the recent reductions in population size estimated for populations with localized distributions (e.g., 7.Inabe‐01; Watanabe and Mori 2012) or those experiencing large fluctuations (e.g., 15.Toyo‐01; Ichiyanagi et al. 2012; Watanabe and Mori 2012) (Figure 3). In particular, population 7.Inabe‐01 nearly disappeared due to disturbances caused by a typhoon in 1990 and subsequent river modifications. An *ex situ* conservation program for this population was initiated in 2003 using a dozen remaining wild individuals as founders. These founders, which were used in this study as 7.Inabe‐01 specimens, exhibited a higher frequency of ROH segments ≥ 1 Mb compared to other populations, likely reflecting recent inbreeding events (Ceballos et al. 2018). This should be carefully considered in ongoing conservation efforts. Since this study relied on samples collected between 1996 and 2009, it is crucial to assess more recent fluctuations in population size and implement the necessary conservation measures based on the latest data.

### 4.2 Population Divergence and Demographic History

Despite the low genetic diversity, genome‐wide SNP data successfully revealed the geographic population structure of *P. ichikawai* , whereas mitogenome and microsatellite data did not. These results highlight the power of genome‐wide SNP data, which provide phylogenetic information from numerous loci, compared to the mitogenome, which represents a single linkage group, and microsatellites, which offer limited phylogenetic information due to their reversible mutation characteristics and are usually restricted to a relatively small number of loci in practice (Edwards et al. 2015).

The *P. ichikawai* populations were primarily divided into two groups, that is, the northern group (populations 5–13) and the eastern (1–4) + western (14–17) group (Figure 2f). The latter group showed the trans‐Ise Bay distribution pattern, that is, a genetically close relationship between populations on the eastern and western sides of the bay. This pattern may reflect the structure of a paleo‐river system in the Ise Bay area (Moriyama 2004). The rCCR calculation and SMC++ analysis suggested that all *P. ichikawai* populations had merged into a single ancestral population by approximately 1.0–3 × 10^4^ years ago, corresponding to the Last Glacial Period, when a paleo‐river system in the Ise Bay area was maximally developed due to the lowered sea level (Moriyama 2004). This paleo‐river system had a large basin encompassing the present Nobi, Ise, and Okazaki plains as well as the entire Ise Bay. The ancestral population in this paleo‐river system likely differentiated genetically into two populations located in the upstream and downstream areas of the plain. This process may explain the trans‐Ise Bay distribution pattern also observed in plain‐dwelling fish species, such as *Pseudorasbora pugnax* and *Hemigrammocypris neglectus* (Cho and Mukai 2023; Watanabe and Mori 2008). The subsequent postglacial sea‐level rise should have led to the population differentiation of *P. ichikawai* , as well as other species, among the present‐day river systems. This process of population differentiation may be characteristic of bay‐like landforms (Watanabe and Nishida 2003; Watanabe and Takahashi 2010). Similar influences of historical sea‐level change and paleo‐drainage structure on freshwater fish phylogeography have also been documented in other bay‐shaped or lowland coastal regions (e.g., Egge and Hagbo 2015; Perea and Doadrio 2015; Thomaz et al. 2017). However, comparative analyses across species remain a subject for future research, and in particular, the timing inferred from mitochondrial DNA alone for other species should be carefully re‐examined.

Genome‐wide SNP data also revealed strong genetic cohesion among populations within each river system, which was not detected by microsatellite data. Neighbor‐net analysis, PopCluster barplots, and PCA and UMAP plots based on MIG‐seq data consistently showed that populations from the same river system tended to cluster together (Figures 2d,f, S5, S6). This pattern is consistent with the ecology of *P. ichikawai* , a strictly freshwater species with limited dispersal ability, for which gene flow is generally confined within the same river system. A remarkable point is that the populations in the Toyo River were clearly differentiated into three groups occurring in different tributaries (Figure 2d). This contrasts with the tendency for genetic cohesion observed among local populations within other river systems. Based on the *f*_3_‐statistics analysis, this pattern was not likely derived from hybridization with populations in neighboring river systems. The Toyo River system is located on the Median Tectonic Line, a major tectonic boundary that is a thousand‐kilometer‐long fault extending across southwest Japan, and has many geological structures such as waterfalls that serve as migration barriers for fish. These structures, as well as man‐made in‐river structures such as dams, may have caused the large population differentiation in the Toyo River system.

In addition to the broad geographic structure described above, both microsatellite and genome‐wide SNP data revealed clear genetic differentiation among tributary populations within each river system (Table S3, Figures S5a, S6). These findings suggest that each tributary population should be regarded as a distinct conservation or management unit for *P. ichikawai* . However, as mentioned above, some of the remaining populations are so seriously threatened that they are on the verge of local extinction. In addition, inbreeding may be progressing excessively in the generally fragmented populations of this species (e.g., population 7.Inabe‐01). In such cases, potential conservation measures could include reinforcement from other populations to promote genetic rescue (Frankham et al. 2010; Tallmon et al. 2004; Whiteley et al. 2015). Even if this measure is adopted, the geographical structure of *P. ichikawai* , that is, the northern, western, and eastern groups, should be carefully considered when selecting the source population.

## 5 Conclusion

More than 1000 genome‐wide SNPs obtained by an easy reduced‐representation genome sequencing method were able to reveal shallow, recent population differentiation patterns in the endangered *Pseudobagrus ichikawai* , which could not be detected by mitogenomes or microsatellites. Demographic estimates based on resequencing data were also useful for inferring inter‐population differentiation and subsequent population‐specific histories. Taken together, this information will allow us to deepen our understanding of population history within geographic and climatic timeframes that have not been fully captured by conventional methods. Our findings highlight that the absence of detectable population structure using traditional markers does not necessarily indicate a genuinely unstructured population or a lack of complex demographic history. The application of genome‐wide SNP data is therefore essential for uncovering cryptic diversity and identifying appropriate conservation units in endangered species, especially when conventional genetic indices provide limited information. This perspective will further facilitate the understanding of regional natural history and the practical conservation of biodiversity.

## Author Contributions

**Keisuke Onuki:** conceptualization (equal), formal analysis (lead), funding acquisition (supporting), investigation (equal), writing – original draft (lead), writing – review and editing (lead). **Ryoichi Tabata:** funding acquisition (supporting), investigation (equal), writing – review and editing (supporting). **Tappei Mishina:** funding acquisition (supporting), investigation (supporting), writing – review and editing (supporting). **Mutsumi Nishida:** conceptualization (supporting), writing – review and editing (supporting). **Katsutoshi Watanabe:** conceptualization (equal), formal analysis (supporting), funding acquisition (lead), investigation (equal), project administration (lead), resources (lead), supervision (lead), writing – original draft (supporting), writing – review and editing (supporting).

## Funding

This work was supported by Environmental Restoration and Conservation Agency, 10.13039/100014423, JPMEERF20224M02. Japan Society for the Promotion of Science, 10.13039/501100001691, 20H03009, 21K14869, 22J23327, 22KJ2004, 25K02327, 97J05164.

## Conflicts of Interest

The authors declare no conflicts of interest.

## Supporting information


**Figure S1:** Hi‐C contact map of the *Pseudobagrus ichikawai* genome. The blocks represent the contact frequencies between genomic loci.
**Figure S2:** Summary of BlobTools analysis of the *Pseudobagrus ichikawai* genome. (a) BlobPlot of the chromosome‐level assembly of *P. ichikawai* , showing GC proportion versus read coverage for each scaffold, along with marginal histograms for GC proportion and coverage. (b) Taxonomic composition of the chromosome‐level assembly, showing the proportions of sequences assigned to different organisms.
**Figure S3:** Correlation matrix among river range and genetic diversity indices of *Pseudobagrus ichikawai* based on three genetic markers: complete mitochondrial genome (mitogenome; approximately 16,530 bp), microsatellites (10 loci), and genome‐wide SNPs (1259 SNPs). Range: the range along the river course (1: ≤ 100 m; 2: < 1 km; 3: < 10 km; 4: ≥ 10 km), *h*: haplotype diversity, *π*: nucleotide diversity, *H*_e_: expected heterozygosity, *N*_e_: contemporary effective population size. The diagonal panels show the density plots of the indices. The upper right of the matrix shows Kendall's rank correlation coefficient (*τ*) and its *p*‐value. Three asterisks indicate *p* < 0.001, two asterisks indicate *p* < 0.01, one asterisk indicates *p* < 0.05, and no asterisks indicate *p* ≥ 0.05.
**Figure S4:** Cumulative fraction of the genome made up of ROHs at least 100 kb long in the genome of *Pseudobagrus ichikawai* .
**Figure S5:** Results of unsupervised clustering for *Pseudobagrus ichikawai* using two genetic markers: microsatellite (10 loci) and genome‐wide SNPs (1259 SNPs). Estimated individual admixture proportions with PopCluster at K = 2 to 17 using (a) microsatellite data and (b) genome‐wide SNP data.
**Figure S6:** Results of principal component analysis (PCA) and uniform manifold approximation and projection (UMAP) for *Pseudobagrus ichikawai* using two genetic markers: microsatellites (10 loci) and genome‐wide SNPs (1259 SNPs). Scatter plots for principal components 1 and 2 using (a) microsatellite data and (b) genome‐wide SNP data. Scatter plots of UMAP results using (c) microsatellite data and (d) genome‐wide SNP data.
**Figure S7:** Estimated split times and 95% confidence intervals for each population pair of *Pseudobagrus ichikawai* using the “split” function implemented in SMC++. The red dots indicate results based on the original data. The 95% confidence intervals were calculated based on 20 bootstrap data sets. The color of the confidence interval bars corresponds to that of the population pairs shown in Figure 3e.

**Table S1:** Sample information of *Pseudobagrus ichikawai* used in the study.
**Table S2:** List of primer sequences used to prepare the MIG‐seq library.
**Table S3:** Results of MIG‐seq and whole‐genome resequencing of *Pseudobagrus ichikawai* used in the study.
**Table S4:** Pairwise genetic differentiation (*F*_ST_) and corresponding *p*‐values among *Pseudobagrus ichikawai* populations based on (a) microsatellite data and (b) MIG‐seq data, demonstrating significant genetic divergence among almost all populations.
**Table S5:** Results of *f*_3_‐test for 17 *Pseudobagrus ichikawai* populations, showing no evidence of admixture among populations after their initial divergence.

**Data S1:** ece373263‐sup‐0003‐Supinfo.txt.

## Data Availability

The genome assembly of *Pseudobagrus ichikawai* is deposited in the DNA Data Bank of Japan (DDBJ) (AP044115–AP044587; BioProject PRJDB35862). Raw sequence reads are deposited in the DDBJ Sequence Read Archive (DRA) (BioProject PRJDB35862). Complete mitogenome sequences are deposited in DDBJ (LC888378–LC888448). Microsatellite genotyping data in GENEPOP format are available as Supporting Information (Data S1). The supplementary figures and tables are provided as Figures S1–S7 and Tables S1–S5. As a guide to the Supporting Information, Figures S1 and S2 provide validation of the genome assembly quality and taxonomic purity. Figure S3 presents a correlation matrix among river ranges and various genetic diversity indices. Figures S4–S6 and Table S4 detail the genomic distribution of ROH, population clustering, and genetic differentiation. Figure S7 and Table S5 show estimated population split times and results of admixture tests. Tables S1–S3 provide sample metadata, primer sequences, and sequencing statistics for MIG‐seq and whole‐genome resequencing.

## References

[ece373263-bib-0001] Allendorf, F. W. , W. C. Funk , S. N. Aitken , M. Byrne , and G. Luikart . 2022. “Conservation Units.” In Conservation and the Genomics of Populations, edited by F. W. Allendorf , W. C. Funk , S. N. Aitken , M. Byrne , G. Luikart , and A. Antunes , 451–486. Oxford University Press. 10.1093/oso/9780198856566.003.0020.

[ece373263-bib-0002] Aquadro, C. F. , and B. D. Greenberg . 1983. “Human Mitochondrial DNA Variation and Evolution: Analysis of Nucleotide Sequences From Seven Individuals.” Genetics 103, no. 2: 287–312. 10.1093/genetics/103.2.287. 6299878 PMC1219980

[ece373263-bib-0003] Avise, J. C. 1994. Molecular Markers, Natural History and Evolution. Springer US. 10.1007/978-1-4615-2381-9.

[ece373263-bib-0004] Avise, J. C. 2000. Phylogeography: The History and Formation of Species. Harvard University Press.

[ece373263-bib-0005] Avise, J. C. , J. Arnold , R. M. Ball , et al. 1987. “Intraspecific Phylogeography: The Mitochondrial DNA Bridge Between Population Genetics and Systematics.” Annual Review of Ecology and Systematics 18: 489–522.

[ece373263-bib-0006] Boitard, S. , W. Rodríguez , F. Jay , S. Mona , and F. Austerlitz . 2016. “Inferring Population Size History From Large Samples of Genome‐Wide Molecular Data—An Approximate Bayesian Computation Approach.” PLoS Genetics 12, no. 3: e1005877. 10.1371/journal.pgen.1005877. 26943927 PMC4778914

[ece373263-bib-0007] Brito, P. H. , and S. V. Edwards . 2009. “Multilocus Phylogeography and Phylogenetics Using Sequence‐Based Markers.” Genetica 135, no. 3: 439–455. 10.1007/s10709-008-9293-3. 18651229

[ece373263-bib-0008] Bronstein, O. , A. Kroh , and E. Haring . 2018. “Mind the Gap! The Mitochondrial Control Region and Its Power as a Phylogenetic Marker in Echinoids.” BMC Evolutionary Biology 18, no. 1: 80. 10.1186/s12862-018-1198-x. 29848319 PMC5977486

[ece373263-bib-0009] Brumfield, R. T. , P. Beerli , D. A. Nickerson , and S. V. Edwards . 2003. “The Utility of Single Nucleotide Polymorphisms in Inferences of Population History.” Trends in Ecology & Evolution 18, no. 5: 249–256. 10.1016/S0169-5347(03)00018-1.

[ece373263-bib-0010] Ceballos, F. C. , P. K. Joshi , D. W. Clark , M. Ramsay , and J. F. Wilson . 2018. “Runs of Homozygosity: Windows Into Population History and Trait Architecture.” Nature Reviews Genetics 19, no. 4: 220–234. 10.1038/nrg.2017.109. 29335644

[ece373263-bib-0011] Chang, C. C. , C. C. Chow , L. C. Tellier , S. Vattikuti , S. M. Purcell , and J. J. Lee . 2015. “Second‐Generation PLINK: Rising to the Challenge of Larger and Richer Datasets.” GigaScience 4, no. 1: 7. 10.1186/s13742-015-0047-8. 25722852 PMC4342193

[ece373263-bib-0012] Chen, S. , Y. Zhou , Y. Chen , and J. Gu . 2018. “fastp: An Ultra‐Fast All‐In‐One FASTQ Preprocessor.” Bioinformatics 34, no. 17: i884–i890. 10.1093/bioinformatics/bty560. 30423086 PMC6129281

[ece373263-bib-0013] Cheng, H. , G. T. Concepcion , X. Feng , H. Zhang , and H. Li . 2021. “Haplotype‐Resolved De Novo Assembly Using Phased Assembly Graphs With Hifiasm.” Nature Methods 18, no. 2: 170–175. 10.1038/s41592-020-01056-5. 33526886 PMC7961889

[ece373263-bib-0014] Cho, H. , and T. Mukai . 2023. “Mitogenomic Phylogeny Revealed the Fine Population Structure of an Endangered Cyprinid Fish *Pseudorasbora pugnax* in the Tokai Region, Central Japan.” Ichthyological Research 70, no. 2: 243–255. 10.1007/s10228-022-00883-0.

[ece373263-bib-0015] Cho, Y. S. , L. Hu , H. Hou , et al. 2013. “The Tiger Genome and Comparative Analysis With Lion and Snow Leopard Genomes.” Nature Communications 4, no. 1: 2433. 10.1038/ncomms3433. PMC3778509 24045858

[ece373263-bib-0016] Danecek, P. , A. Auton , G. Abecasis , et al. 2011. “The Variant Call Format and VCFtools.” Bioinformatics 27, no. 15: 2156–2158. 10.1093/bioinformatics/btr330. 21653522 PMC3137218

[ece373263-bib-0017] Danecek, P. , J. K. Bonfield , J. Liddle , et al. 2021. “Twelve Years of SAMtools and BCFtools.” GigaScience 10, no. 2: giab008. 10.1093/gigascience/giab008. 33590861 PMC7931819

[ece373263-bib-0018] Delaneau, O. , B. Howie , A. J. Cox , J.‐F. Zagury , and J. Marchini . 2013. “Haplotype Estimation Using Sequencing Reads.” American Journal of Human Genetics 93, no. 4: 687–696. 10.1016/j.ajhg.2013.09.002. 24094745 PMC3791270

[ece373263-bib-0019] Delaneau, O. , J. Marchini , and J.‐F. Zagury . 2012. “A Linear Complexity Phasing Method for Thousands of Genomes.” Nature Methods 9, no. 2: 179–181. 10.1038/nmeth.1785. 22138821

[ece373263-bib-0020] Do, C. , R. S. Waples , D. Peel , G. M. Macbeth , B. J. Tillett , and J. R. Ovenden . 2014. “NeEstimator v2: Re‐Implementation of Software for the Estimation of Contemporary Effective Population Size (*N* _e_) From Genetic Data.” Molecular Ecology Resources 14, no. 1: 209–214. 10.1111/1755-0998.12157. 23992227

[ece373263-bib-0021] Dobrynin, P. , S. Liu , G. Tamazian , et al. 2015. “Genomic Legacy of the African Cheetah, *Acinonyx jubatus* .” Genome Biology 16, no. 1: 277. 10.1186/s13059-015-0837-4. 26653294 PMC4676127

[ece373263-bib-0022] Dudchenko, O. , S. S. Batra , A. D. Omer , et al. 2017. “De Novo Assembly of the *Aedes aegypti* Genome Using Hi‐C Yields Chromosome‐Length Scaffolds.” Science 356, no. 6333: 92–95. 10.1126/science.aal3327. 28336562 PMC5635820

[ece373263-bib-0023] Durand, N. C. , J. T. Robinson , M. S. Shamim , et al. 2016. “Juicebox Provides a Visualization System for Hi‐C Contact Maps With Unlimited Zoom.” Cell Systems 3, no. 1: 99–101. 10.1016/j.cels.2015.07.012. 27467250 PMC5596920

[ece373263-bib-0024] Durand, N. C. , M. S. Shamim , I. Machol , et al. 2016. “Juicer Provides a One‐Click System for Analyzing Loop‐Resolution Hi‐C Experiments.” Cell Systems 3, no. 1: 95–98. 10.1016/j.cels.2016.07.002. 27467249 PMC5846465

[ece373263-bib-0025] Edwards, S. V. , V. V. Robin , N. Ferrand , and C. Moritz . 2022. “The Evolution of Comparative Phylogeography: Putting the Geography (And More) Into Comparative Population Genomics.” Genome Biology and Evolution 14, no. 1: evab176. 10.1093/gbe/evab176. 34347070 PMC8743039

[ece373263-bib-0026] Edwards, S. V. , A. J. Shultz , and S. C. Campbell‐Staton . 2015. “Next‐Generation Sequencing and the Expanding Domain of Phylogeography.” Folia Zoologica 64, no. 3: 187–206. 10.25225/fozo.v64.i3.a2.2015.

[ece373263-bib-0027] Egge, J. J. D. , and T. J. Hagbo . 2015. “Comparative Phylogeography of Mississippi Embayment Fishes.” PLoS One 10, no. 3: e0116719. 10.1371/journal.pone.0116719. 25826456 PMC4380359

[ece373263-bib-0028] Excoffier, L. , and H. E. L. Lischer . 2010. “Arlequin Suite Ver 3.5: A New Series of Programs to Perform Population Genetics Analyses Under Linux and Windows.” Molecular Ecology Resources 10, no. 3: 564–567. 10.1111/j.1755-0998.2010.02847.x. 21565059

[ece373263-bib-0029] Frankham, R. , J. D. Ballou , and D. A. Briscoe . 2010. Introduction to Conservation Genetics. 2nd ed. Cambridge University Press. 10.1017/CBO9780511809002.

[ece373263-bib-0030] Fraser, D. J. , and L. Bernatchez . 2001. “Adaptive Evolutionary Conservation: Towards a Unified Concept for Defining Conservation Units.” Molecular Ecology 10, no. 12: 2741–2752. 10.1046/j.0962-1083.2001.01411.x. 11903888

[ece373263-bib-0031] Goldstein, D. B. , A. Ruiz Linares , L. L. Cavalli‐Sforza , and M. W. Feldman . 1995. “Genetic Absolute Dating Based on Microsatellites and the Origin of Modern Humans.” Proceedings of the National Academy of Sciences 92, no. 15: 6723–6727. 10.1073/pnas.92.15.6723. PMC41401 7624310

[ece373263-bib-0032] Hashiguchi, Y. , T. Mishina , H. Takeshima , et al. 2024. “Draft Genome of Akame (*Lates japonicus*) Reveals Possible Genetic Mechanisms for Long‐Term Persistence and Adaptive Evolution With Low Genetic Diversity.” Genome Biology and Evolution 16, no. 8: evae174. 10.1093/gbe/evae174. 39109913 PMC11346364

[ece373263-bib-0033] Hewitt, G. M. 2004a. “Genetic Consequences of Climatic Oscillations in the Quaternary.” Philosophical Transactions: Biological Sciences 359, no. 1442: 183–195. 15101575 10.1098/rstb.2003.1388 PMC1693318

[ece373263-bib-0034] Hewitt, G. M. 2004b. “The Structure of Biodiversity—Insights From Molecular Phylogeography.” Frontiers in Zoology 1, no. 1: 4. 10.1186/1742-9994-1-4. 15679920 PMC544936

[ece373263-bib-0035] Huson, D. H. , and D. Bryant . 2006. “Application of Phylogenetic Networks in Evolutionary Studies.” Molecular Biology and Evolution 23, no. 2: 254–267. 10.1093/molbev/msj030. 16221896

[ece373263-bib-0036] Ichiyanagi, H. , K. Watanabe , and S. Mori . 2012. “Habitats and Population Dynamics of the Bagrid Catfish *Pseudobagrus ichikawai* .” Ecology and Civil Engineering 15, no. 2: 257–267. 10.3825/ece.15.257.

[ece373263-bib-0037] International Union for Conservation of Nature . 2022. “The IUCN Red List of Threatened Species. Version 2022–2.” https://www.iucnredlist.org.

[ece373263-bib-0038] Ito, T. , K. Hosoya , and J.‐I. Miyazaki . 2019. “ *Lefua tokaiensis*, a New Species of Nemacheilid Loach From Central Japan (Teleostei: Nemacheilidae).” Ichthyological Research 66, no. 4: 479–487. 10.1007/s10228-019-00690-0.

[ece373263-bib-0039] Iwasaki, W. , T. Fukunaga , R. Isagozawa , et al. 2013. “MitoFish and MitoAnnotator: A Mitochondrial Genome Database of Fish With an Accurate and Automatic Annotation Pipeline.” Molecular Biology and Evolution 30, no. 11: 2531–2540. 10.1093/molbev/mst141. 23955518 PMC3808866

[ece373263-bib-0040] Japan Ministry of the Environment . 2020. “Publication of the Japanese Red List 2020.” https://www.env.go.jp/press/107905.html.

[ece373263-bib-0041] Jin, J.‐J. , W.‐B. Yu , J.‐B. Yang , et al. 2020. “GetOrganelle: A Fast and Versatile Toolkit for Accurate De Novo Assembly of Organelle Genomes.” Genome Biology 21, no. 1: 241. 10.1186/s13059-020-02154-5. 32912315 PMC7488116

[ece373263-bib-0042] Kadota, M. , O. Nishimura , H. Miura , K. Tanaka , I. Hiratani , and S. Kuraku . 2020. “Multifaceted Hi‐C Benchmarking: What Makes a Difference in Chromosome‐Scale Genome Scaffolding?” GigaScience 9, no. 1: giz158. 10.1093/gigascience/giz158. 31919520 PMC6952475

[ece373263-bib-0043] Kawase, S. , and K. Hosoya . 2015. “ *Pseudorasbora pugnax*, a New Species of Minnow From Japan, and Redescription of *P. pumila* (Teleostei: Cyprinidae).” Ichthyological Exploration of Freshwaters 25, no. 4: 289–298.

[ece373263-bib-0044] Korunes, K. L. , and K. Samuk . 2021. “Pixy: Unbiased Estimation of Nucleotide Diversity and Divergence in the Presence of Missing Data.” Molecular Ecology Resources 21, no. 4: 1359–1368. 10.1111/1755-0998.13326. 33453139 PMC8044049

[ece373263-bib-0045] Laetsch, D. R. , and M. L. Blaxter . 2017. “BlobTools: Interrogation of Genome Assemblies.” F1000Research 6: 1287. 10.12688/f1000research.12232.1.

[ece373263-bib-0046] Li, H. 2011. “A Statistical Framework for SNP Calling, Mutation Discovery, Association Mapping and Population Genetical Parameter Estimation From Sequencing Data.” Bioinformatics 27, no. 21: 2987–2993. 10.1093/bioinformatics/btr509. 21903627 PMC3198575

[ece373263-bib-0047] Li, H. , and R. Durbin . 2011. “Inference of Human Population History From Individual Whole‐Genome Sequences.” Nature 475, no. 7357: 493–496. 10.1038/nature10231. 21753753 PMC3154645

[ece373263-bib-0048] Lisiecki, L. E. , and M. E. Raymo . 2005. “A Pliocene‐Pleistocene Stack of 57 Globally Distributed Benthic δ^18^O Records.” Paleoceanography 20: PA1003. 10.1029/2004PA001071.

[ece373263-bib-0049] Maier, R. , P. Flegontov , O. Flegontova , U. Işıldak , P. Changmai , and D. Reich . 2023. “On the Limits of Fitting Complex Models of Population History to F‐Statistics.” eLife 12: e85492. 10.7554/eLife.85492. 37057893 PMC10310323

[ece373263-bib-0050] Malinsky, M. , H. Svardal , A. M. Tyers , et al. 2018. “Whole‐Genome Sequences of Malawi Cichlids Reveal Multiple Radiations Interconnected by Gene Flow.” Nature Ecology & Evolution 2, no. 12: 1940–1955. 10.1038/s41559-018-0717-x. 30455444 PMC6443041

[ece373263-bib-0051] Manni, M. , M. R. Berkeley , M. Seppey , F. A. Simão , and E. M. Zdobnov . 2021. “BUSCO Update: Novel and Streamlined Workflows Along With Broader and Deeper Phylogenetic Coverage for Scoring of Eukaryotic, Prokaryotic, and Viral Genomes.” Molecular Biology and Evolution 38, no. 10: 4647–4654. 10.1093/molbev/msab199. 34320186 PMC8476166

[ece373263-bib-0052] Marske, K. A. , C. Rahbek , and D. Nogués‐Bravo . 2013. “Phylogeography: Spanning the Ecology‐Evolution Continuum.” Ecography 36, no. 11: 1169–1181. 10.1111/j.1600-0587.2013.00244.x.

[ece373263-bib-0053] McCormack, J. E. , S. M. Hird , A. J. Zellmer , B. C. Carstens , and R. T. Brumfield . 2013. “Applications of Next‐Generation Sequencing to Phylogeography and Phylogenetics.” Molecular Phylogenetics and Evolution 66, no. 2: 526–538. 10.1016/j.ympev.2011.12.007. 22197804

[ece373263-bib-0054] McInnes, L. , J. Healy , and J. Melville . 2020. “UMAP: Uniform Manifold Approximation and Projection for Dimension Reduction.” (arXiv:1802.03426) arXiv. 10.48550/arXiv.1802.03426.

[ece373263-bib-0055] Meirmans, P. G. , and P. H. Van Tienderen . 2004. “GENOTYPE and GENODIVE: Two Programs for the Analysis of Genetic Diversity of Asexual Organisms.” Molecular Ecology Notes 4, no. 4: 792–794. 10.1111/j.1471-8286.2004.00770.x.

[ece373263-bib-0056] Mikheenko, A. , V. Saveliev , P. Hirsch , and A. Gurevich . 2023. “WebQUAST: Online Evaluation of Genome Assemblies.” Nucleic Acids Research 51, no. W1: W601–W606. 10.1093/nar/gkad406. 37194696 PMC10320133

[ece373263-bib-0057] Mizuno, H. , K. Nakayama , T. Akita , Y. Hashiguchi , T. Osugi , and H. Takeshima . 2025. “Detailed Kinship Estimation for Detecting Bias Among Breeding Families in a Reintroduced Population of the Endangered Bagrid Catfish *Tachysurus ichikawai* .” Population Ecology 67, no. 2: 109–124. 10.1002/1438-390X.12183.

[ece373263-bib-0058] Mori, S. , and M. Nagoshi . 2001. “Nekogigi.” In Freshwater Fishes of Japan, edited by H. Kawanabe , N. Mizuno , and K. Hosoya , 3rd ed., 408–409. Yama‐kei Publishers.

[ece373263-bib-0059] Morin, P. A. , G. Luikart , R. K. Wayne , and the SNP workshop group . 2004. “SNPs in Ecology, Evolution and Conservation.” Trends in Ecology & Evolution 19, no. 4: 208–216. 10.1016/j.tree.2004.01.009.

[ece373263-bib-0060] Moriyama, A. 2004. “Submarine Topography, Especially the Formation of Caldrons and Sand Banks in the Ise and Mikawa Bay.” Bulletin of Aichi University of Education, Natural Sciences 53: 39–56.

[ece373263-bib-0061] Nadachowska‐Brzyska, K. , M. Konczal , and W. Babik . 2022. “Navigating the Temporal Continuum of Effective Population Size.” Methods in Ecology and Evolution 13, no. 1: 22–41. 10.1111/2041-210X.13740.

[ece373263-bib-0062] Nakajima, J. 2012. “Taxonomic Study of the *Cobitis striata* Complex (Cypriniformes, Cobitidae) in Japan.” Zootaxa 3586, no. 1: 1. 10.11646/zootaxa.3586.1.11.

[ece373263-bib-0063] Nater, A. , R. Burri , T. Kawakami , L. Smeds , and H. Ellegren . 2015. “Resolving Evolutionary Relationships in Closely Related Species With Whole‐Genome Sequencing Data.” Systematic Biology 64, no. 6: 1000–1017. 10.1093/sysbio/syv045. 26187295 PMC4604831

[ece373263-bib-0064] Okada, Y. , and S. S. Kubota . 1957. “Description of a New Fresh Water Cat‐Fish, *Coreobagrus ichikawai*, With an Emendation of the Genus *Coreobagrus* MORI.” Japanese Journal of Ichthyology 5, no. 3–6: 143–145. 10.11369/jji1950.5.143.

[ece373263-bib-0065] Onuki, K. , and Y. Fuke . 2022. “Rediscovery of a Native Freshwater Shrimp, *Neocaridina denticulata*, and Expansion of an Invasive Species in and Around Lake Biwa, Japan: Genetic and Morphological Approach.” Conservation Genetics 23, no. 5: 967–980. 10.1007/s10592-022-01467-1.

[ece373263-bib-0066] Patterson, N. , P. Moorjani , Y. Luo , et al. 2012. “Ancient Admixture in Human History.” Genetics 192, no. 3: 1065–1093. 10.1534/genetics.112.145037. 22960212 PMC3522152

[ece373263-bib-0067] Perea, S. , and I. Doadrio . 2015. “Phylogeography, Historical Demography and Habitat Suitability Modelling of Freshwater Fishes Inhabiting Seasonally Fluctuating Mediterranean River Systems: A Case Study Using the Iberian Cyprinid *Squalius Valentinus* .” Molecular Ecology 24, no. 14: 3706–3722. 10.1111/mec.13274. 26085305

[ece373263-bib-0068] Pockrandt, C. , M. Alzamel , C. S. Iliopoulos , and K. Reinert . 2020. “GenMap: Ultra‐Fast Computation of Genome Mappability.” Bioinformatics 36, no. 12: 3687–3692. 10.1093/bioinformatics/btaa222. 32246826 PMC7320602

[ece373263-bib-0069] Rochette, N. C. , A. G. Rivera‐Colón , and J. M. Catchen . 2019. “Stacks 2: Analytical Methods for Paired‐End Sequencing Improve RADseq‐Based Population Genomics.” Molecular Ecology 28, no. 21: 4737–4754. 10.1111/mec.15253. 31550391

[ece373263-bib-0070] Schiffels, S. , and R. Durbin . 2014. “Inferring Human Population Size and Separation History From Multiple Genome Sequences.” Nature Genetics 46, no. 8: 919–925. 10.1038/ng.3015. 24952747 PMC4116295

[ece373263-bib-0071] Schiffels, S. , and K. Wang . 2020. “MSMC and MSMC2: The Multiple Sequentially Markovian Coalescent.” In Statistical Population Genomics, edited by J. Y. Dutheil , 147–166. Springer US. 10.1007/978-1-0716-0199-0_7. 31975167

[ece373263-bib-0072] Suyama, Y. , and Y. Matsuki . 2015. “MIG‐Seq: An Effective PCR‐Based Method for Genome‐Wide Single‐Nucleotide Polymorphism Genotyping Using the Next‐Generation Sequencing Platform.” Scientific Reports 5, no. 1: 16963. 10.1038/srep16963. 26593239 PMC4655332

[ece373263-bib-0073] Suzuki, T. , S. Kimura , and K. Shibukawa . 2019. “Two New Lentic, Dwarf Species of *Rhinogobius* Gill, 1859 (Gobiidae) From Japan.” Bulletin of the Kanagawa Prefectural Museum (Natural Science) 2019, no. 48: 21–36. 10.32225/bkpmnh.2019.48_21.

[ece373263-bib-0074] Takezaki, N. , M. Nei , and K. Tamura . 2014. “POPTREEW: Web Version of POPTREE for Constructing Population Trees From Allele Frequency Data and Computing Some Other Quantities.” Molecular Biology and Evolution 31, no. 6: 1622–1624. 10.1093/molbev/msu093. 24603277

[ece373263-bib-0075] Tallmon, D. A. , G. Luikart , and R. S. Waples . 2004. “The Alluring Simplicity and Complex Reality of Genetic Rescue.” Trends in Ecology & Evolution 19, no. 9: 489–496. 10.1016/j.tree.2004.07.003. 16701312

[ece373263-bib-0076] Terhorst, J. , J. A. Kamm , and Y. S. Song . 2017. “Robust and Scalable Inference of Population History From Hundreds of Unphased Whole Genomes.” Nature Genetics 49, no. 2: 303–309. 10.1038/ng.3748. 28024154 PMC5470542

[ece373263-bib-0077] Thomaz, A. T. , L. R. Malabarba , and L. L. Knowles . 2017. “Genomic Signatures of Paleodrainages in a Freshwater Fish Along the Southeastern Coast of Brazil: Genetic Structure Reflects Past Riverine Properties.” Heredity 119, no. 4: 287–294. 10.1038/hdy.2017.46. 28767104 PMC5597787

[ece373263-bib-0078] Ueno, K. 1985. “Karyotype of the Japanese and Korean Bagrid Fishes.” Kaiyo Kagaku 17: 102–108.

[ece373263-bib-0079] Vasimuddin, M. , S. Misra , H. Li , and S. Aluru . 2019. “Efficient Architecture‐Aware Acceleration of BWA‐MEM for Multicore Systems.” 2019IEEE International Parallel and Distributed Processing Symposium (IPDPS), 314–324. 10.1109/IPDPS.2019.00041.

[ece373263-bib-0080] Wang, J. 2002. “An Estimator for Pairwise Relatedness Using Molecular Markers.” Genetics 160, no. 3: 1203–1215. 10.1093/genetics/160.3.1203. 11901134 PMC1462003

[ece373263-bib-0081] Wang, J. 2022. “Fast and Accurate Population Admixture Inference From Genotype Data From a Few Microsatellites to Millions of SNPs.” Heredity 129, no. 2: 79–92. 10.1038/s41437-022-00535-z. 35508539 PMC9338324

[ece373263-bib-0082] Watanabe, K. 1994a. “Growth, Maturity and Population Structure of the Bagrid Catfish, *Pseudobagrus ichikawai*, in the Tagiri River, Mie Prefecture, Japan.” Japanese Journal of Ichthyology 41, no. 1: 15–22. 10.11369/jji1950.41.15.

[ece373263-bib-0083] Watanabe, K. 1994b. “Mating Behavior and Larval Development of *Pseudobagrus ichikawai* (Siluriformes: Bagridae).” Japanese Journal of Ichthyology 41, no. 3: 243–251. 10.11369/jji1950.41.243.

[ece373263-bib-0084] Watanabe, K. 2008. “Diel Activity and Reproductive Territory of the Japanese Bagrid Catfish, *Pseudobagrus ichikawai* .” Environmental Biology of Fishes 81, no. 1: 77–86. 10.1007/s10641-006-9173-6.

[ece373263-bib-0085] Watanabe, K. , and S. Mori . 2008. “Comparison of Genetic Population Structure Between Two Cyprinids, *Hemigrammocypris rasborella* and *Pseudorasbora pumila* Subsp., in the Ise Bay Basin, Central Honshu, Japan.” Ichthyological Research 55, no. 4: 309–320. 10.1007/s10228-008-0038-1.

[ece373263-bib-0086] Watanabe, K. , and S. Mori . 2012. “ *Pseudobagrus ichikawai*: Toward Active Conservation Strategies.” Japanese Journal of Ichthyology 59, no. 2: 168–171. 10.11369/jji.59.168.

[ece373263-bib-0087] Watanabe, K. , and M. Nishida . 2003. “Genetic Population Structure of Japanese Bagrid Catfishes.” Ichthyological Research 50, no. 2: 140–148. 10.1007/s10228-002-0149-z.

[ece373263-bib-0088] Watanabe, K. , and H. Takahashi . 2010. “Towards a Deeper Understanding of the Japanese Freshwater Fish Fauna and Its Formation Process.” In Tansuigyorui Chiri no Shizenshi (Natural History of Freshwater Fish Geography), edited by K. Watanabe and H. Takahashi , 217–238. Hokkaido University Press.

[ece373263-bib-0089] Watanabe, K. , K. Tominaga , J. Nakajima , R. Kakioka , and R. Tabata . 2017. “Japanese Freshwater Fishes: Biogeography and Cryptic Diversity.” In Species Diversity of Animals in Japan, edited by M. Motokawa and H. Kajihara , 183–227. Springer Japan. 10.1007/978-4-431-56432-4_7.

[ece373263-bib-0090] Watanabe, K. , T. Watanabe , and M. Nishida . 2001. “Isolation and Characterization of Microsatellite Loci From the Endangered Bagrid Catfish, *Pseudobagrus ichikawai* .” Molecular Ecology Notes 1, no. 1–2: 61–63. 10.1046/j.1471-8278.2000.00024.x.

[ece373263-bib-0091] Wenger, A. M. , P. Peluso , W. J. Rowell , et al. 2019. “Accurate Circular Consensus Long‐Read Sequencing Improves Variant Detection and Assembly of a Human Genome.” Nature Biotechnology 37, no. 10: 1155–1162. 10.1038/s41587-019-0217-9. PMC6776680 31406327

[ece373263-bib-0092] Whiteley, A. R. , S. W. Fitzpatrick , W. C. Funk , and D. A. Tallmon . 2015. “Genetic Rescue to the Rescue.” Trends in Ecology & Evolution 30, no. 1: 42–49. 10.1016/j.tree.2014.10.009. 25435267

[ece373263-bib-0093] Xue, Y. , J. Prado‐Martinez , P. H. Sudmant , et al. 2015. “Mountain Gorilla Genomes Reveal the Impact of Long‐Term Population Decline and Inbreeding.” Science 348, no. 6231: 242–245. 10.1126/science.aaa3952. 25859046 PMC4668944

[ece373263-bib-0094] Zhang, D.‐X. , and G. M. Hewitt . 2003. “Nuclear DNA Analyses in Genetic Studies of Populations: Practice, Problems and Prospects.” Molecular Ecology 12, no. 3: 563–584. 10.1046/j.1365-294X.2003.01773.x. 12675814

[ece373263-bib-0095] Zink, R. M. 2010. “Drawbacks With the Use of Microsatellites in Phylogeography: The Song Sparrow *Melospiza melodia* as a Case Study.” Journal of Avian Biology 41, no. 1: 1–7. 10.1111/j.1600-048X.2009.04903.x.

