# Supplementary material for: Genome‐Wide Analysis Successfully Resolves Population Structure Shaped by Recent Divergence in the Endangered Bagrid Catfish Pseudobagrus ichikawai
Source: Ecol Evol. 2026 Mar 26;16(4):e73263. doi: 10.1002/ece3.73263 (PMC13107278; doi:10.1002/ece3.73263)
Supplement: Supplementary file 1 — Figure S1: Hi‐C contact map of the Pseudobagrus ichikawai genome. The blocks represent the contact frequencies between genomic loci. Figure S2: Summary of BlobTools analysis of the Pseudobagrus ichikawai genome. (a) BlobPlot of the chromosome‐level assembly of P. ichikawai , showing GC proportion versus read coverage for each scaffold, along with marginal histograms for GC proportion and coverage. (b) Taxonomic composition of the chromosome‐level assembly, showing the proportions of sequences assigned to different organisms. Figure S3: Correlation matrix among river range and genetic diversity indices of Pseudobagrus ichikawai based on three genetic markers: complete mitochondrial genome (mitogenome; approximately 16,530 bp), microsatellites (10 loci), and genome‐wide SNPs (1259 SNPs). Range: the range along the river course (1: ≤ 100 m; 2: < 1 km; 3: < 10 km; 4: ≥ 10 km), h: haplotype diversity, π: nucleotide diversity, H e: expected heterozygosity, N e: contemporary effective population size. The diagonal panels show the density plots of the indices. The upper right of the matrix shows Kendall's rank correlation coefficient (τ) and its p‐value. Three asterisks indicate p < 0.001, two asterisks indicate p < 0.01, one asterisk indicates p < 0.05, and no asterisks indicate p ≥ 0.05. Figure S4: Cumulative fraction of the genome made up of ROHs at least 100 kb long in the genome of Pseudobagrus ichikawai . Figure S5: Results of unsupervised clustering for Pseudobagrus ichikawai using two genetic markers: microsatellite (10 loci) and genome‐wide SNPs (1259 SNPs). Estimated individual admixture proportions with PopCluster at K = 2 to 17 using (a) microsatellite data and (b) genome‐wide SNP data. Figure S6: Results of principal component analysis (PCA) and uniform manifold approximation and projection (UMAP) for Pseudobagrus ichikawai using two genetic markers: microsatellites (10 loci) and genome‐wide SNPs (1259 SNPs). Scatter plots for principal components 1 and 2 using [file ECE3-16-e73263-s001.pdf]

Supporting information 1

Supplementary Figures

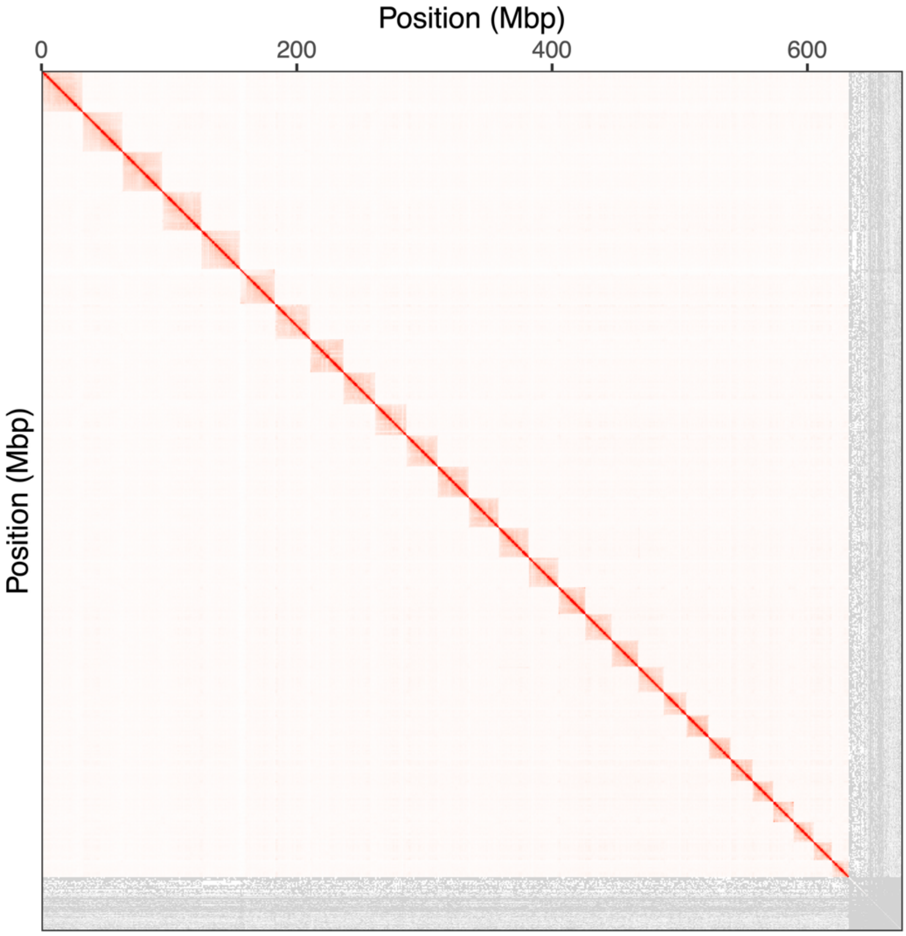

Figure S1: Hi-C contact map of the *Pseudobagrus ichikawai* genome. The frequency of Hi-C interaction links is color coded, ranging from white (low) to red (high).

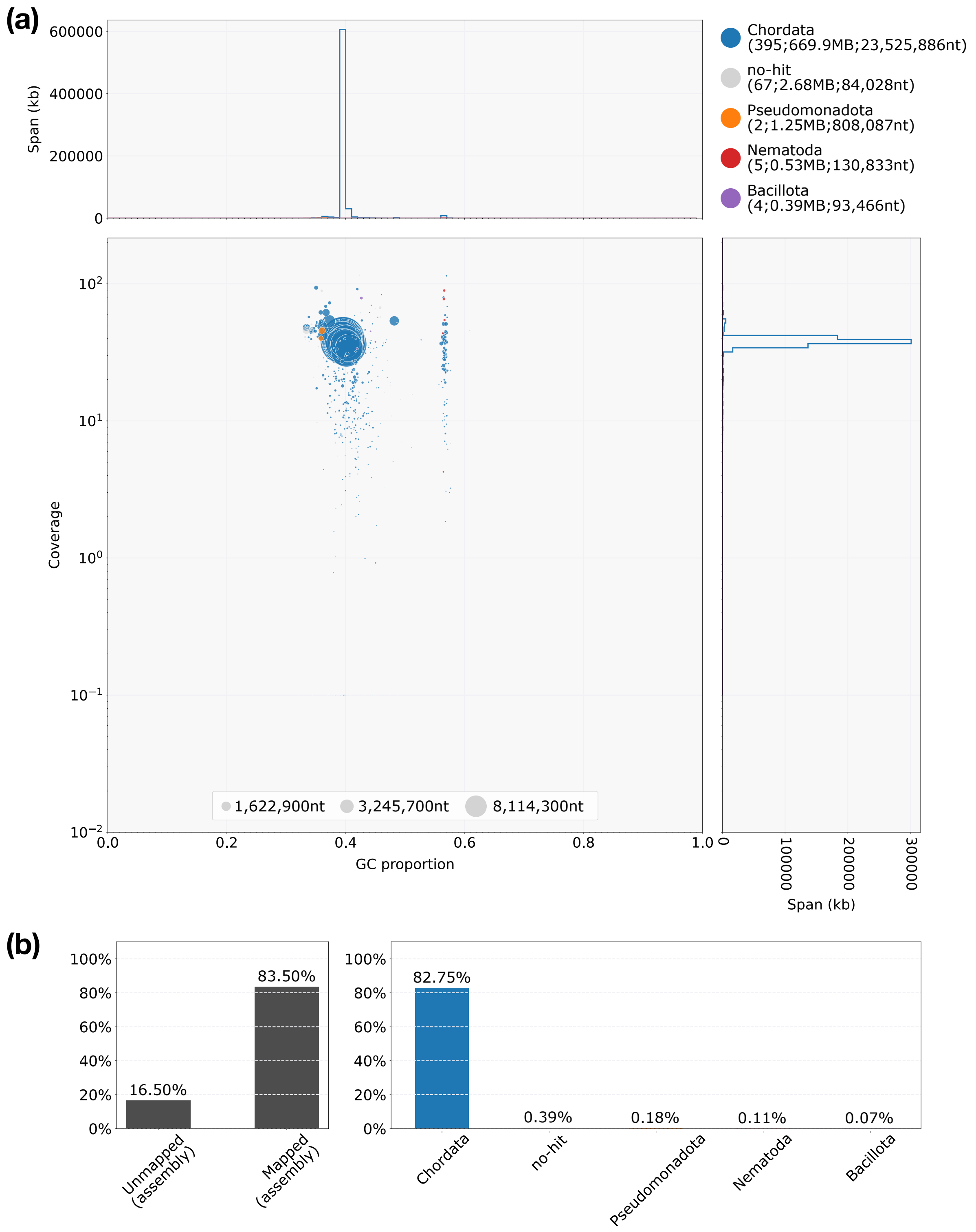

Figure S2: Summary of BlobTools analysis of the *Pseudobagrus ichikawai* genome. (a) BlobPlot of the chromosome-level assembly of *P. ichikawai*, showing GC proportion versus read coverage for each scaffold, along with marginal histograms for GC proportion and coverage. (b) Taxonomic composition of the chromosome-level assembly, showing the proportions of sequences assigned to different organisms.

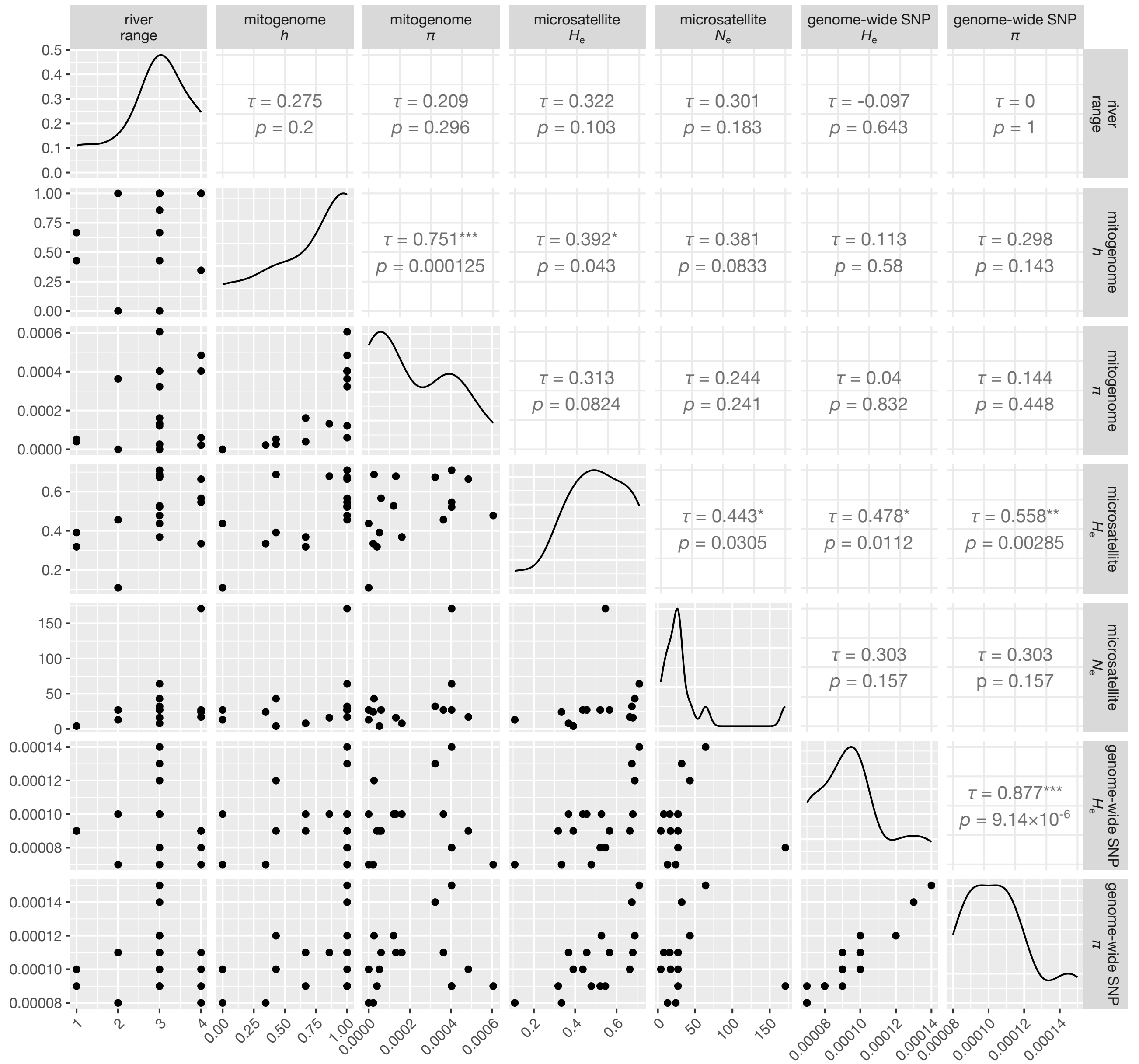

Figure S3: Correlation matrix among river range and genetic diversity indices of *Pseudobagrus ichikawai* based on three genetic markers: complete mitochondrial genome (mitogenome; approximately 16,530 bp), microsatellites (10 loci), and genome-wide SNPs (1,259 SNPs). Range: the range along the river course (1: ≤100 m; 2: < 1 km; 3: <10 km; 4: ≥10 km), *h*: haplotype diversity, *π*: nucleotide diversity, H_e: expected heterozygosity, N_e: contemporary effective population size. The diagonal panels show density plots displaying the distribution of each index. The upper right of the matrix shows Kendall's rank correlation coefficient (τ) and its *p*-value. Three asterisks indicate $p < 0.001$, two asterisks indicate $p < 0.01$, one asterisk indicates $p < 0.05$, and no asterisks indicate $p \geq 0.05$.

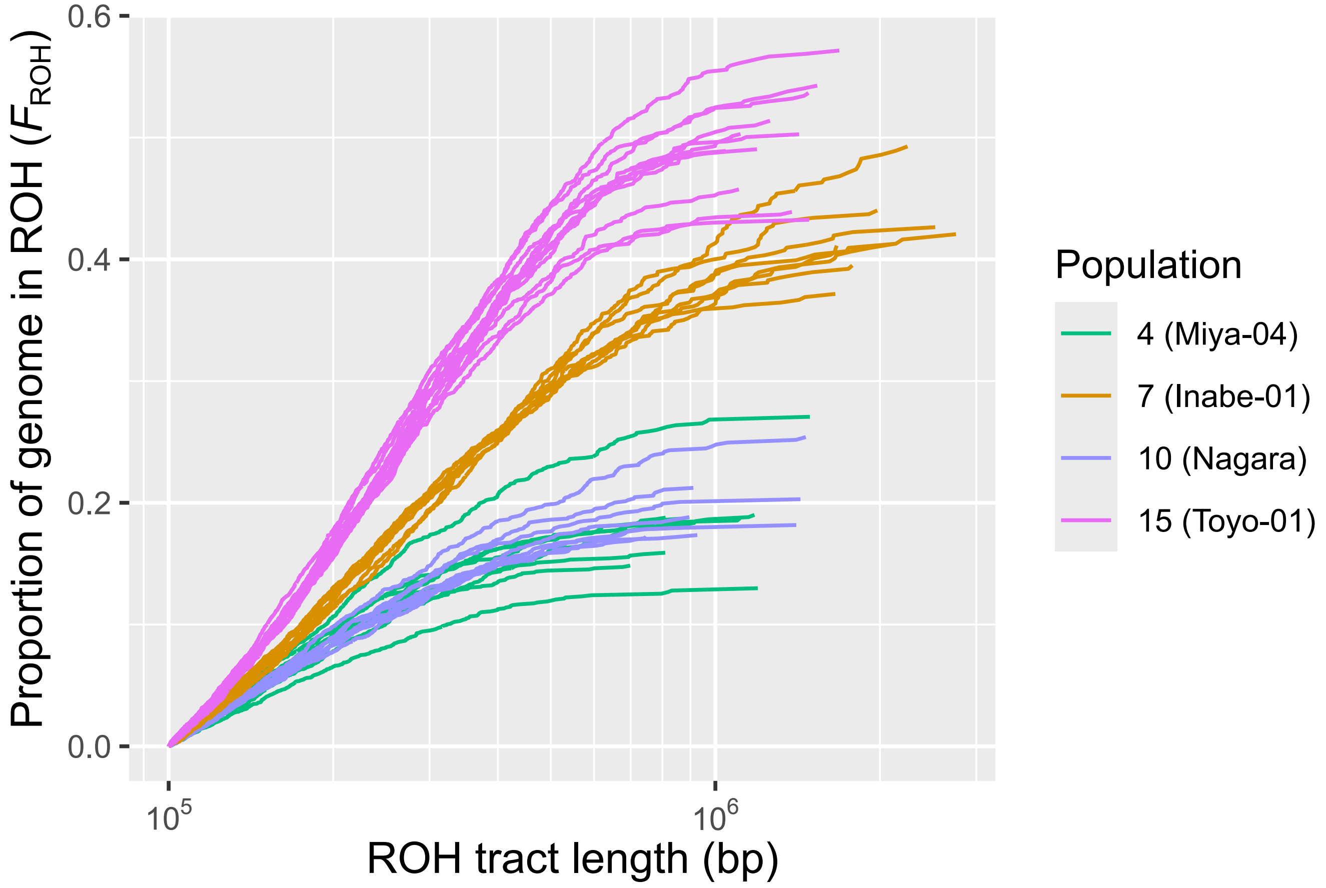

Figure S4: Cumulative fraction of the genome made up of ROHs at least 100 kb long in the genome of *Pseudobagrus ichikawai*.

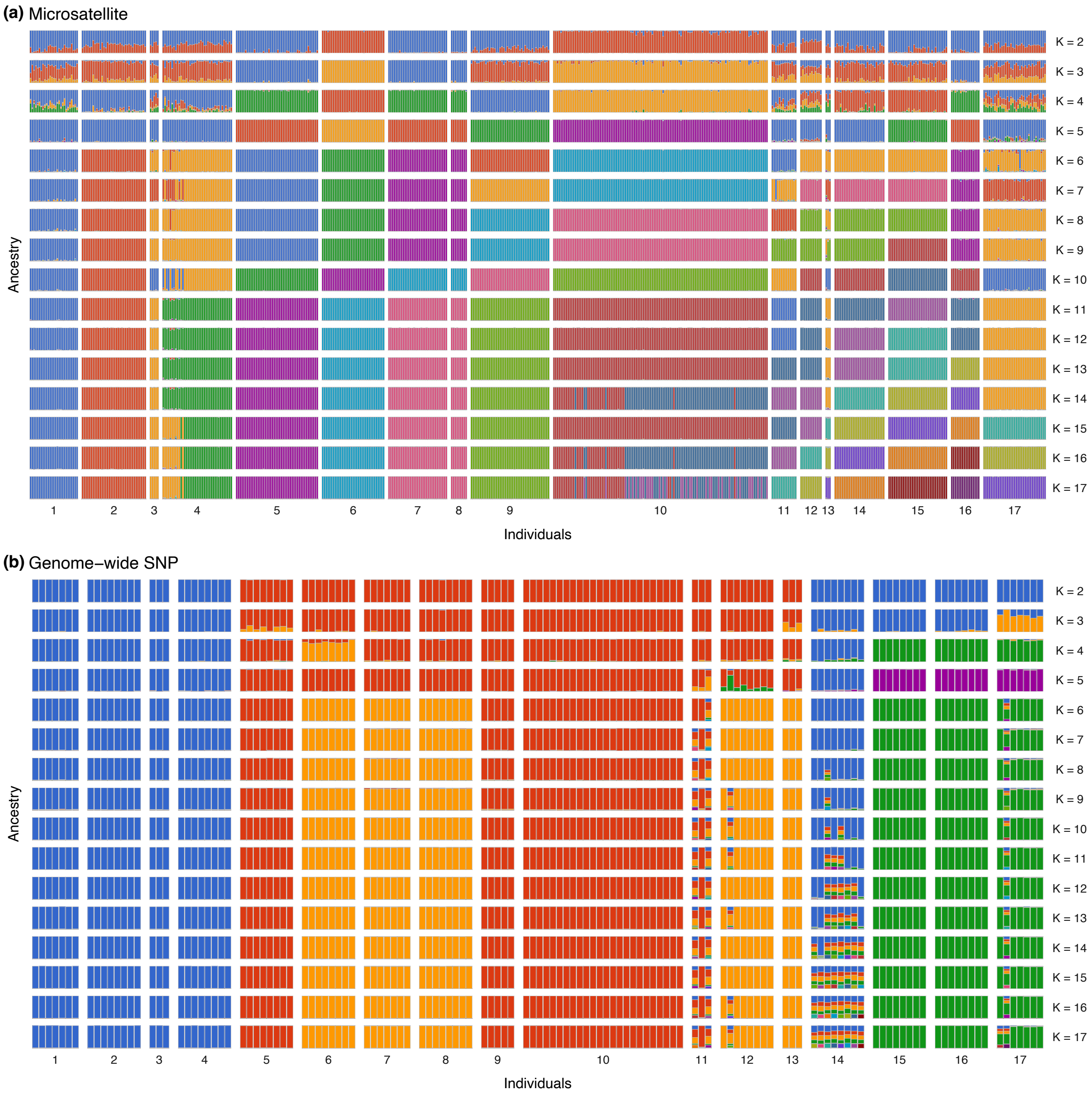

Figure S5: Results of unsupervised clustering for *Pseudobagrus ichikawai* using two genetic markers: microsatellite (10 loci) and genome-wide SNPs (1,259 SNPs). Estimated individual admixture proportions with PopCluster at $K = 2$ to 17 using (a) microsatellite data and (b) genome-wide SNP data.

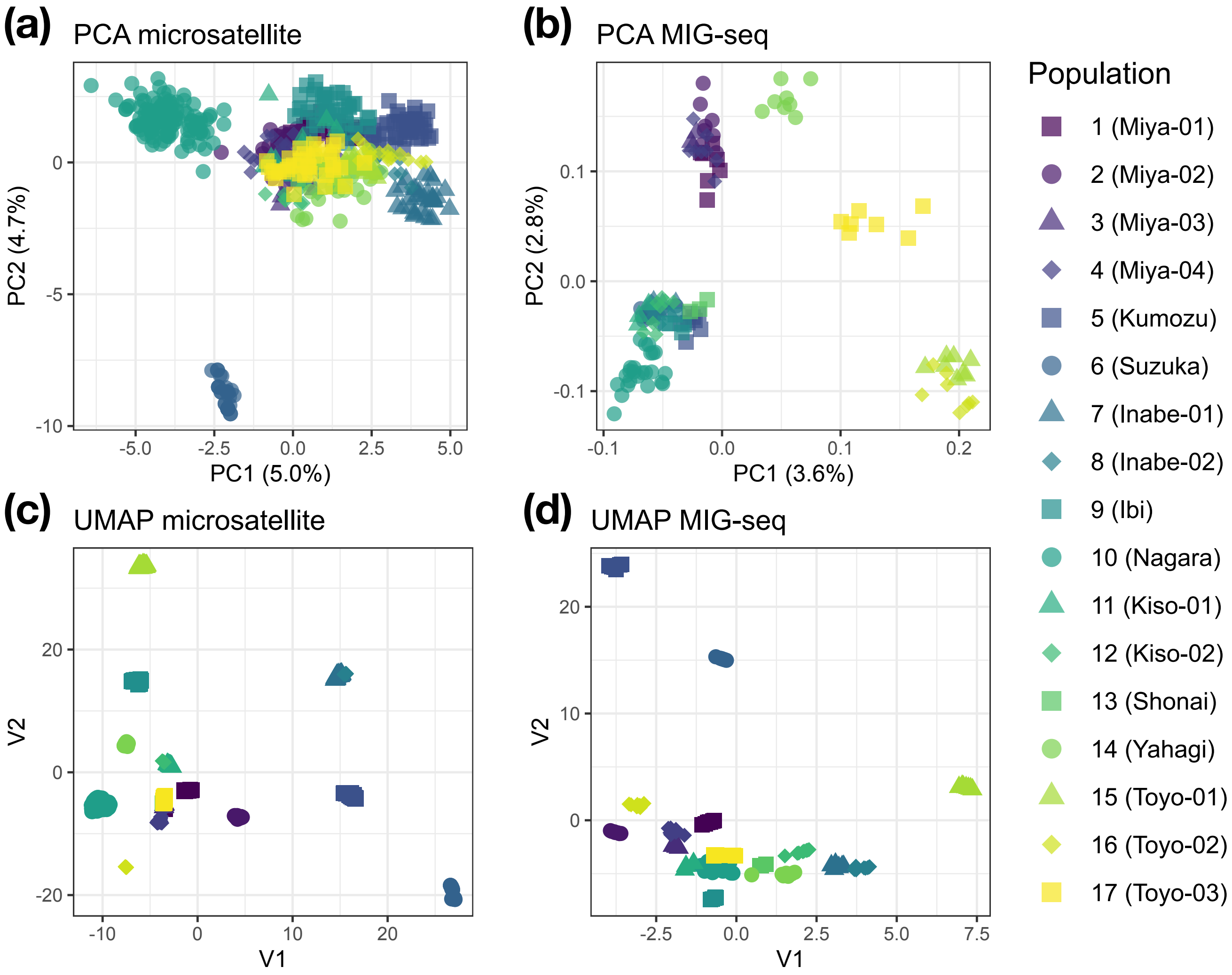

Figure S6: Results of principal component analysis (PCA) and uniform manifold approximation and projection (UMAP) for *Pseudobagrus ichikawai* using two genetic markers: microsatellites (10 loci) and genome-wide SNPs (1,259 SNPs). Scatter plots for principal components 1 and 2 using (a) microsatellite data and (b) genome-wide SNP data. Scatter plots of UMAP results using (c) microsatellite data and (d) genome-wide SNP data.

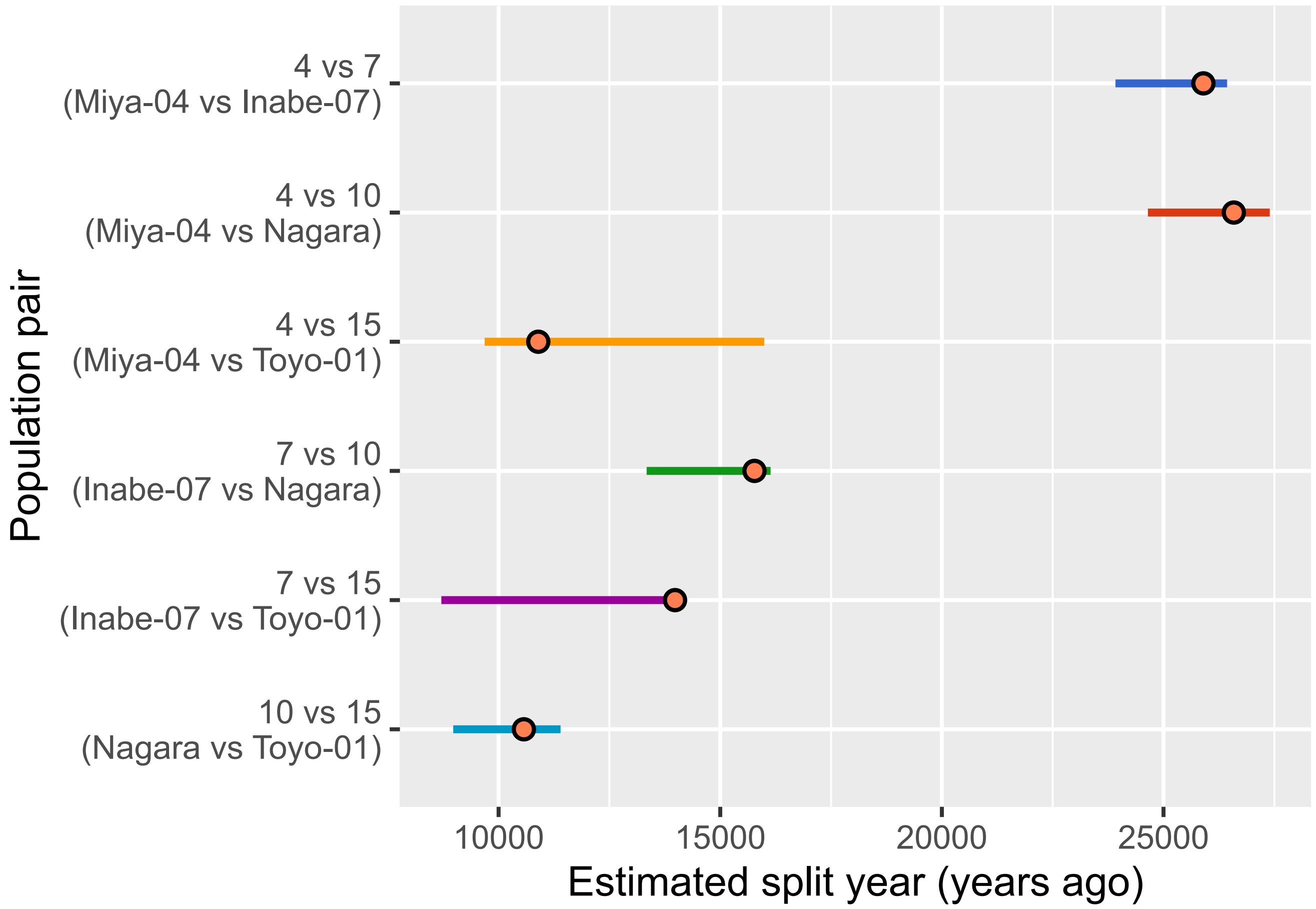

Figure S7: Estimated split times and 95% confidence intervals for each population pair of *Pseudobagrus ichikawai* using the 'split' function implemented in SMC++. The red dots indicate results based on the original data. The 95% confidence intervals were calculated based on 20 bootstrap datasets. The color of the confidence interval bars corresponds to that of the population pairs shown in Figure 3e.
